# Urinary extracellular vesicles: A position paper by the Urine Task Force of the International Society for Extracellular Vesicles

**DOI:** 10.1002/jev2.12093

**Published:** 2021-05-21

**Authors:** Uta Erdbrügger, Charles J. Blijdorp, Irene V. Bijnsdorp, Francesc E. Borràs, Dylan Burger, Benedetta Bussolati, James Brian Byrd, Aled Clayton, James W. Dear, Juan M. Falcón‐Pérez, Cristina Grange, Andrew F. Hill, Harry Holthöfer, Ewout J. Hoorn, Guido Jenster, Connie R. Jimenez, Kerstin Junker, John Klein, Mark A. Knepper, Erik H. Koritzinsky, James M. Luther, Metka Lenassi, Janne Leivo, Inge Mertens, Luca Musante, Eline Oeyen, Maija Puhka, Martin E. van Royen, Catherine Sánchez, Carolina Soekmadji, Visith Thongboonkerd, Volkert van Steijn, Gerald Verhaegh, Jason P. Webber, Kenneth Witwer, Peter S.T. Yuen, Lei Zheng, Alicia Llorente, Elena S. Martens‐Uzunova

**Affiliations:** ^1^ Division of Nephrology University of Virginia Health System Charlottesville Virginia USA; ^2^ Department of Internal medicine Division of Nephrology and Transplantation Erasmus MC University Medical Center Rotterdam Rotterdam The Netherlands; ^3^ Department of Molecular Cell Biology Institute for Cancer Research Oslo University Hospital Oslo Norway; ^4^ Department for Mechanical Electronics and Chemical Engineering Oslo Metropolitan University Oslo Norway; ^5^ Erasmus MC Cancer Institute Department of Urology Laboratory of Experimental Urology University Medical Center Rotterdam Erasmus MC Rotterdam The Netherlands; ^6^ Department of Urology Amsterdam UMC location VUmc Cancer Center Amsterdam Amsterdam The Netherlands; ^7^ REMAR‐IVECAT Group, “Germans Trias i Pujol” Health Science Research Institute and Nephrology Department “Germans Trias i Pujol” University Hospital Badalona Spain; ^8^ Kidney Research Centre Ottawa Hospital Research Institute and Department of Cellular and Molecular Medicine University of Ottawa Canada; ^9^ Department of Molecular Biotechnology and Health Science University of Torino Torino Italy; ^10^ Department of Internal Medicine University of Michigan Medical School Ann Arbor Michigan USA; ^11^ Tissue Microenvironment Group Division of Cancer & Genetics School of Medicine Cardiff University United Kingdom; ^12^ Centre for Cardiovascular Science Queen's Medical Research Institute University of Edinburgh Edinburgh United Kingdom; ^13^ Exosomes Laboratory and Metabolomics Platform Bizkaia Technology Park CIC bioGUNE‐BRTA Derio Spain; ^14^ Department of Medical Sciences University of Torino Torino Italy; ^15^ Department of Biochemistry and Genetics, La Trobe Institute for Molecular Science La Trobe University Bundoora Victoria Australia; ^16^ Internal Medicine University Clinic Hamburg‐Eppendorf Hamburg Germany; ^17^ Institute for Molecular Medicine Finland FIMM University of Helsinki Helsinki Finland; ^18^ Department of Urology Saarland University Medical Centre Homburg Germany; ^19^ Department of Medicine University of Louisville School of Medicine Louisville Kentucky USA; ^20^ Epithelial Systems Biology Laboratory Systems Biology Center National Heart, Lung, and Blood Institute National Institutes of Health Bethesda Maryland USA; ^21^ Renal Diagnostics and Therapeutics Unit National Institute of Diabetes and Digestive and Kidney Diseases National Institutes of Health Bethesda Maryland USA; ^22^ Division of Clinical Pharmacology Department of Medicine Vanderbilt University Medical Center Nashville Tennessee USA; ^23^ Institute of Biochemistry Faculty of Medicine University of Ljubljana Ljubljana Slovenia; ^24^ Department of Biochemistry University of Turku Turku Finland; ^25^ Centre for Proteomics, University of Antwerp and Health Unit, VITO Antwerp Belgium; ^26^ Department of Pathology Erasmus MC Cancer Institute University Medical Center Rotterdam Erasmus MC Rotterdam The Netherlands; ^27^ Department of Urology Clínica las Condes Research Core Santiago Chile; ^28^ Department of Cell and Molecular Biology QIMR Berghofer Medical Research Institute Brisbane Australia; ^29^ Medical Proteomics Unit Office for Research and Development Faculty of Medicine Siriraj Hospital Mahidol University Bangkok Thailand; ^30^ Department of Chemical Engineering TU Delft Delft The Netherlands; ^31^ Department of Urology Radboud University Medical Center Nijmegen The Netherlands; ^32^ Institute of Life Science 1 Swansea University Medical School Singleton Park Campus Swansea UK; ^33^ Departments of Molecular and Comparative Pathobiology and Neurology John Hopkins University School of Medicine Baltimore Maryland USA; ^34^ Department of Laboratory Medicine Nanfang Hospital Southern Medical University Guangzhou Guangdong China

**Keywords:** biobank, biomarkers, bladder, extracellular vesicles, kidney, liquid biopsy, prostate, rigor and standardization, urine

## Abstract

Urine is commonly used for clinical diagnosis and biomedical research. The discovery of extracellular vesicles (EV) in urine opened a new fast‐growing scientific field. In the last decade urinary extracellular vesicles (uEVs) were shown to mirror molecular processes as well as physiological and pathological conditions in kidney, urothelial and prostate tissue. Therefore, several methods to isolate and characterize uEVs have been developed. However, methodological aspects of EV separation and analysis, including normalization of results, need further optimization and standardization to foster scientific advances in uEV research and a subsequent successful translation into clinical practice. This *position paper* is written by the Urine Task Force of the Rigor and Standardization Subcommittee of ISEV consisting of nephrologists, urologists, cardiologists and biologists with active experience in uEV research. Our aim is to present the *state of the art* and identify challenges and gaps in current uEV‐based analyses for clinical applications. Finally, recommendations for improved rigor, reproducibility and interoperability in uEV research are provided in order to facilitate advances in the field.

## 1 INTRODUCTION

Urinalysis has been part of standard clinical practice since antiquity (Magiorkinis & Diamantis, 2015). Today, urine is the second most commonly used biofluid for clinical diagnostics after blood. Urine is produced by the kidneys to eliminate waste products (e.g., urea, metabolites) from the body and to maintain the homeostasis of water, ions, and pH in blood. Humans normally generate approximately 1–2 liters of urine per day, which is released via the urinary tract (ureters, urinary bladder, and urethra). In addition to soluble components like organic and inorganic molecules, urine typically contains some epithelial and blood cells, bacteria, viruses and importantly also extracellular vesicles (EVs) (Pisitkun et al., 2004; Santiago‐Rodriguez et al., 2015). One key advantage of working with urine compared to other biofluids is that it can be easily and frequently collected in large quantities in a noninvasive manner (Decramer et al., 2008; Harpole et al., 2016). However, urinary concentration and contents are highly variable and of dynamic nature due to differences in fluid intake, time of collection, diet and exercise, age, gender, medications, and health status. These well recognized factors can complicate data interpretation and the use of urine in diagnostics, particularly when reference normality ranges are to be set (Guo et al., 2015; Molina et al., 2011; Nagaraj & Mann, 2011; Parolini et al., 2009). These variables may be equally relevant for uEV analyses, and hence lessons from other fields employing urine analysis are likely to be important and applicable for uEV research.

**FIGURE 1:**
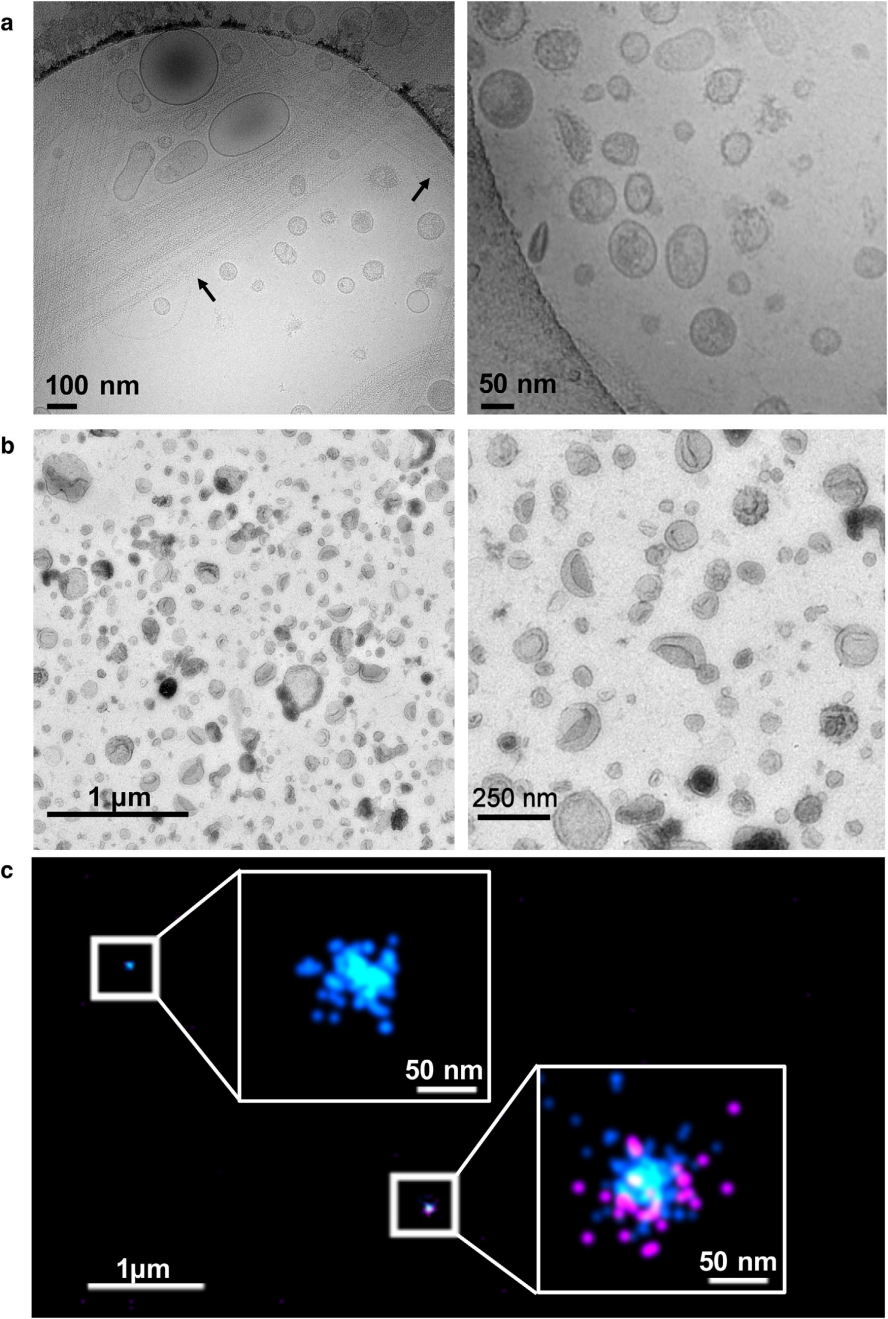
uEV microscopy. (a) Urinary EVs (uEVs) were isolated by centrifugation (20,000 × *g* pellet) and processed for cryoelectron microscopy (as described in (Musante et al., 2020)). The left image shows a wide variety of EVs in size, density and shape. In addition, polymers of uromodulin are shown which seem to entrap uEVs (see arrows). The right image shows a higher magnification of uEVs demonstrating spike like structures emerging from the phospholipid bilayer which likely represents the glycocalyx of some uEVs. (b) uEVs were isolated with ultracentrifugation (100,000 × *g* pellet) and processed for transmission electron microscopy (TEM) using a negative staining protocol (as described in (Puhka et al., 2017)). To the left there is a lower magnification image displaying a large number and variety of uEVs in size, shape and density. The right image shows a higher magnification demonstrating the uEV heterogeneity with differential staining densities and some spike like surface features that can be visualized despite the cup shape morphology which is due to the processing of TEM. (c) Super‐resolution images were obtained using a Nanoimager S Mark II microscope from ONI (Oxford Nanoimaging) equipped with 405 nm/150 mW, 473 nm/1 W, 560 nm/1 W, 640 nm/1 W lasers and dual emission channels split at 640 nm. The figure shows uEVs stained for CD81 (cyan) and Klotho (magenta) using primary antibodies conjugated with Alexa Fluor 555 and 647 respectively. Representative images with zoomed in insets show the expression and nanoscale distribution of the peptide and tetraspanin on the surface of two representative EVs bound to the coverslip surface. Two‐channel dSTORM data was acquired sequentially at 30 Hz in total internal reflection fluorescence (TIRF) mode. Single molecule data was filtered using NimOS (Version 1.7.1.10213, ONI) based on point spread function shape, photon count and localization precision to minimize background noise and remove low precision localizations

The presence of EVs in urine was first documented by electron microscopy images in 1986 when Wiggins *et al*. investigated the procoagulant activity of pelletable material (100,000 × *g* ultracentrifugation) in normal urine (Wiggins et al., 1986). Representative examples for images of EVs including electron microscopy are shown in Figure 1. Several years later, membrane vesicles of tubular (100,000 × *g* pellet) (Scherberich, 1989) and podocyte (200,000 × *g* pellet) (Pascual et al., 1994) origin were described in urine from patients with glomerulonephritis. However, uEVs caught wider attention in 2004 when Pisitkun *et al*. provided a thorough characterization of uEVs pelleted by ultracentrifugation of urine at 200,000 × *g* (Pisitkun et al., 2004). In this pioneering mass spectrometry analysis, the authors identified 295 proteins including typical proteins originating from nephron epithelial cells and urothelial cells, as well as proteins involved in the formation of multivesicular bodies. This initial overview of the proteome of uEVs and the evident alteration of the molecular composition of uEVs in pathological conditions opened a new frontier of biomarker discovery, sparking an exponential growth in uEV research and providing new possibilities for the use of urine in noninvasive clinical diagnostics. Urinary EV isolates enabled the detection of molecules that were not previously identified in urine because of their low concentration in the bulk fluid or because of their location inside EVs. Importantly, many of these low concentration proteins are connected to specific cells and/or organs (Gonzales et al., 2009; Santucci et al., 2015).

**FIGURE 2:**
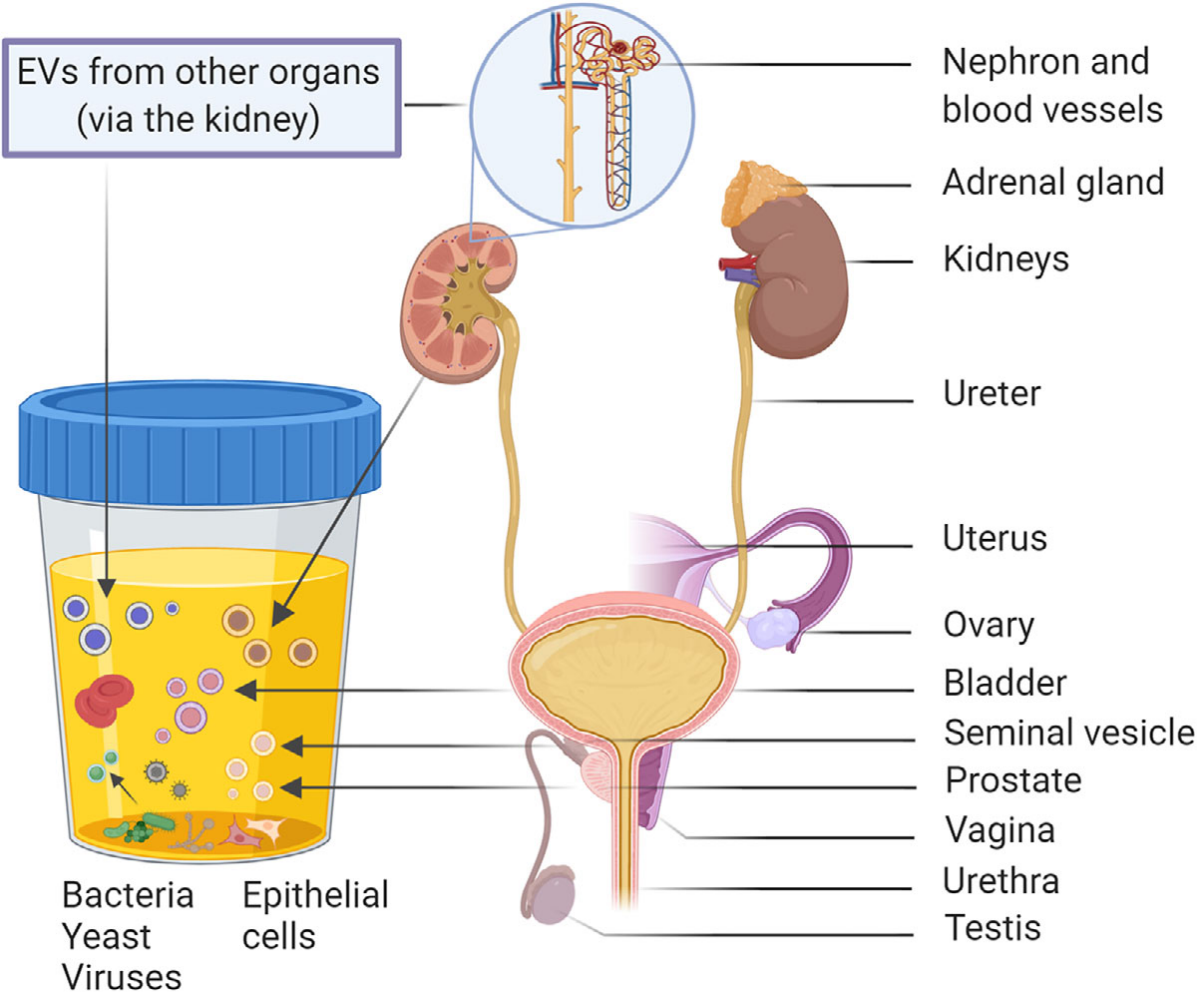
Origins of urinary EVs

Urinary EVs have generally been considered to originate from cells of the urogenital tract and the residing bacteria and may be mixed with similarly‐sized viruses (Figure 2). Therefore, uEVs constitute a source of potential molecular biomarkers for diseases of the kidneys, bladder and urogenital tract (prostate, uterus/vagina), and likely play a functional role in the physiology and pathology of these organs (Erdbrugger & Le, 2016; Karpman et al., 2017; Merchant et al., 2017). Importantly, however, proteins arising from other distant anatomical sites in the body have also been identified in uEVs. For example, uEVs have been proposed as a source of biomarkers for diseases such as Parkinson's disease and lung cancer (Fraser et al., 2016; Li et al., 2011). Nevertheless, analysis of uEVs may open a window into the EV‐repertoire of the circulation and provide a systemic readout of disease states from a non‐invasive sample.

Both standard analytical methods and high‐throughput omics technologies have been applied in (urinary) EV biomarker research, leading to the discovery of numerous potential EV‐based biomarkers for a range of diseases. Early studies focused mainly on cancers related to the urogenital system and led to the identification of protein, mRNA, miRNA, lipid and metabolite biomarkers for prostate, bladder, and renal cancers (Bijnsdorp et al., 2013; Chen et al., 2012; Clos‐Garcia et al., 2018; Del Boccio et al., 2012; Dhondt et al., 2018; Fujita et al., 2017; Koppers‐Lalic et al., 2016; Lee et al., 2018; Leiblich, 2017; Mitchell et al., 2009; Nilsson et al., 2009; Øverbye et al., 2015; Raimondo et al., 2013; Rodriguez et al., 2017; Sequeiros et al., 2017; Skotland et al., 2017; Zhan et al., 2018). In particular, two prostate‐associated RNAs, PCA3, and TMPRSS2:ERG, were identified in urinary extracellular vesicles by Nilsson et al. in 2019 (Nilsson et al., 2009). These results were the foundation for a prostate cancer diagnostic test that has been extensively validated in two prospective multi‐center US studies (McKiernan et al., 2016; McKiernan et al., 2018). Altogether, these promising results inspired the search for uEV‐based biomarkers for other urogenital tract pathologies such as polycystic kidney disease, cystinuria, diabetic nephropathy, acute kidney injury/ renal ischemia‐reperfusion injury, glomerulonephritis, renal interstitial fibrosis/ chronic kidney disease, lupus nephritis, nephronophthisis‐related ciliopathies, tubulopathies and primary and secondary hypertension (Abe et al., 2018; Bourderioux et al., 2015; Chun‐Yan et al., 2018; Corbetta et al., 2015; Gonzalez‐Calero et al., 2017; Kwon et al., 2017; La Salvia et al., 2020; Morikawa et al., 2018; Qi et al., 2016; Raimondo et al., 2016; Raimondo et al., 2020; Salih et al., 2016; Salih et al., 2018; Sonoda et al., 2009; Stokman et al., 2019; Tangtanatakul et al., 2018; van der Lubbe et al., 2012; Williams et al., 2020; Wolley et al., 2017; Zubiri et al., 2014). Many of the newly identified candidate biomarkers have not yet been validated in large independent cohorts or in additional laboratories, but nevertheless these examples highlight the enormous potential for uEV analyses as readouts for pathophysiological alterations within the urogenital and other systems.

The diverse origins and dynamic molecular composition of uEVs present an enormous analytical challenge. It is therefore unlikely that a single standardized approach for urine collection, uEV isolation, and measurement will effectively cover all disease scenarios and questions. Nevertheless, arriving at a consensus on best methodological practices is of particular importance in preclinical and clinical uEV studies addressing biomarker discovery and validation, where new understanding would ultimately be applied to inform clinical decisions. Herein, we give a brief overview of the state of the art in uEV research and identify the critical knowledge gaps. We also provide recommendations regarding biospecimen handling, processing, and reporting requirements to improve experimental reproducibility and interoperability. This is of utmost importance for the development of high quality, multi‐site studies and realization of the true potential of uEVs in varied clinical settings.

## 2 BIOLOGY OF URINARY EVs

### 2.1 Origins of uEVs

**FIGURE 3:**
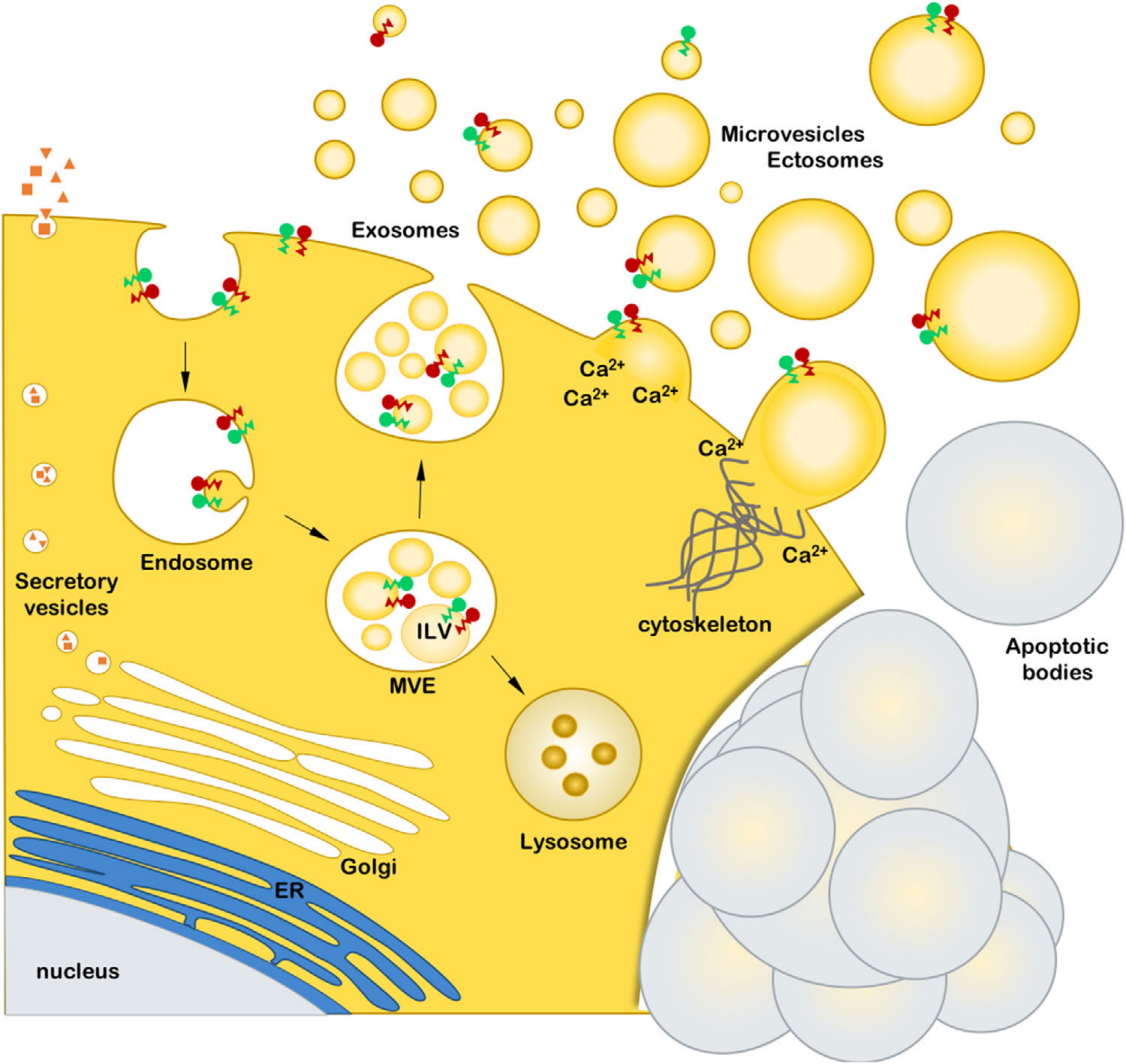
Biogenesis pathways of urinary extracellular vesicles (uEVs). EVs are a highly heterogeneous group of membrane‐bound particles released by both healthy and malignant cells. Generation of exosomes, a specific population of small uEVs, occurs via formation and maturation of multivesicular endosomes (MVEs). Exosomes are formed as intraluminal vesicles (ILVs) in the lumen of MVEs by inward budding of the endosomal membrane. Upon fusion with the cell membrane, exosomes are released into the intercellular space. Microvesicles and ectosomes represent both small and large EVs and are formed by outward budding and scission of the plasma membrane. The process is associated with the accumulation of Ca2^+^‐dependent enzymes that change the polarity of membrane phospholipids. This causes physical bending of the cellular membrane and rearrangements in the underlying cytoskeleton, leading to the formation of microvesicles. Once released by the cell, small uEVs formed at the PM and MVB‐derived exosomes exhibit overlapping size and composition, which makes it difficult to establish their biosynthetic origin. Apoptotic bodies are formed during apoptosis (programed cell death) when cells undergo characteristic outward blebbing caused by breaks in the cytoskeleton. During this process the cellular membrane bulges outward and portions of the cytoplasm and its contents separate forming apoptotic bodies. Secretory vesicles (SV) are produced by the ER and Golgi apparatus. Most of them have specialized cargo such as hormones and neurotransmitters. SVs fuse with the cell membrane at specialized supramolecular structures (porosomes) to release their cargo in the extracellular space.

Urine contains a mixture of EVs that originate from several parts of the urogenital tract, including the kidneys, bladder, prostate (males), and utero‐vaginal tract (females) (Table 1 and Figure 2) (Gonzales et al., 2009; Pisitkun et al., 2004; Zaichick, 2014). The biogenesis of this heterogeneous EV population including exosomes, microvesicles, apoptotic bodies, is illustrated in Figure 3 and discussed in detail in other review papers (Kalluri & LeBleu, 2020; van Niel et al., 2018). The relative contributions of each part of the urogenital tract to the total population of uEVs has not yet been determined, but it has been shown that specific subpopulations of uEVs in urine can be enriched by particular interventions, for example, the collection of urine after digital rectal examination (DRE) increases the amount of prostatic fluid in urine and subsequently the quantity of EVs originating from prostatic luminal epithelium cells (Duijvesz et al., 2015; Hendriks et al., 2016). Hence, it is possible to manipulate the uEV composition in this and perhaps other ways, in order to facilitate the detection of specific uEV‐associated molecules.

**TABLE 1:** List of uEV markers characterizing different structures of the urinary tract

Organ	Structure/cell of origin	EV marker	References
Kidney	Glomerulus (podocytes)	Podocin	(Hogan et al., 2014)
		Podocalyxin	(Hogan et al., 2014)
		Wilms' tumour 1 (WT 1)	(Kalani et al., 2013)
		Complement receptor 1 (CR1)	(Prunotto et al., 2013)
		Canonical transient receptor potential 6 (TRPC6)	(Hogan et al., 2014)
		Nephrin	(Hogan et al., 2014)
	Glomerulus/proximal tubules	Angiotensin‐converting enzyme (ACE)	(Pisitkun et al., 2004)
	Proximal tubules	Megalin	(Pisitkun et al., 2004)
		Aminopeptidase N (APN)	(Pisitkun et al., 2004)
		Cubilin	(Hogan et al., 2014)
		Sodium/glucose cotransporter 2 (SGLT 2)	(Øverbye et al., 2015)
		Carbonic anhydrase (CA IV)	(Pisitkun et al., 2004)
		Na^+^/H^+^ exchanger isoform 3 (NHE3)	(Zhou et al., 2006)
	Renal progenitor cells	CD133 (Prominin 1)	(Dimuccio et al., 2014)
	Tubular epithelial cells	CD24	(Keller et al., 2007)
	Proximal tubules/Henle's loop	Aquaporin 1 (AQP1)	(Pisitkun et al., 2004)
	Henle's loop	Uromodulin (UMOD, Tamm‐Horsfall Protein, THP)	(Pisitkun et al., 2004)
		Na‐K‐2Cl cotransporter (NKCC2)	(Pisitkun et al., 2004)
	Proximal/distal tubules	Klotho	(Grange et al., 2020)
	Distal tubules	Prominin 2	(Turco et al., 2016)
		Thiazide‐sensitive Na‐Cl cotransporter (NCC)	(Pisitkun et al., 2004)
	Distal tubules/collecting duct	Aquaporin 2 (AQP2)	(Pisitkun et al., 2004)
		Claudin 1	(Turco et al., 2016)
	Collecting duct	Mucin‐1	(Pisitkun et al., 2004)
Bladder	Transitional epithelial cells	Uroplakin‐1	(Pisitkun et al., 2004)
		Uroplakin‐2	(Pisitkun et al., 2004)
		Mucin‐1 (MUC‐1)	(Pisitkun et al., 2004)
Prostate	Epithelial cells	Prostatic acid phosphatase (PPAP)	(Øverbye et al., 2015)
		Prostate transglutaminase (TGM4)	(Sequeiros et al., 2017)
		Prostate‐specific membrane antigen (PSMA)	(Mitchell et al., 2009)

The markers were described in uEVs isolated from human urine and identified by Western blot and/or flow cytometric analyses.

Apart from being produced by different cell types in the urogenital tract, uEVs can also originate from residing immune cells, bacteria, and yeast, while enveloped viruses, themselves a type of EV, may also be present (Hiemstra et al., 2014; Nolte‐’t Hoen et al., 2016; Salih et al., 2016; van Dongen et al., 2016). In addition, some reports suggest that a subset of uEVs enters the urine from the circulation and contain many immunity‐related proteins (Erozenci et al., 2019; Oosthuyzen et al., 2016). It is unclear how these EVs reach the urine (Cheng et al., 2012; Erozenci et al., 2019). In order to pass the glomerular filtration barrier (GFB) and basement membrane of the kidney the EVs would have to be smaller than the membrane‐pores (6 nm in the healthy state), or the integrity of the membrane‐pores would need to be perturbed (something seen in various pathological states), allowing passage of larger structures like EVs from the circulation into the urinary space (Longmire et al., 2008; Patrakka et al., 2002). Larger pores of the slit diaphragm of up to 70 nm in size are found in minimal change disease, an example of a proteinuric disease state with podocyte damage. Small EVs are likely able to move through this barrier in this disease state. In addition, the endothelial barrier of the GFB might also be penetrated as it has fenestrae of up to 100 nm in size which can also allow EVs to move through the GFB (Ndisang, 2018). Alternatively, it is possible that uEVs preparations include non‐vesicular circulating proteins. It is likely that these are endocytosed from the blood by renal tubular cells as it has been demonstrated for modified circulating albumin molecules in diabetes (Londono & Bendayan, 2005). The proteins are then released into the urinary space within EVs. This is supported also by proteomic data, in the case of albumin it is shown that uEVs contain this protein (Musante et al., 2020). Similar mechanisms have been described as early as 1989 suggesting that EVs might be transported by transcytosis through podocytes and secreted into the luminal side as ‘waste’ (Kerjaschki et al., 1989).

An additional enigmatic particle type, known as nanobacteria or calcifying nanoparticles (Yaghobee et al., 2015) is discussed controversially. These entities are composed of crystalline minerals, nucleic acids, and other organic material and appear to be replication competent, albeit through ill‐defined processes. Nanobacteria have been associated with various diseases like nephrolithiasis, polycystic kidney diseases, chronic prostatitis, and pelvic pain syndrome (Ciftcioglu et al., 1999; Hjelle et al., 2000; Shoskes et al., 2005). It remains unclear to what extent these structures contribute to the uEV pool. Further, EVs from both Gram‐positive and Gram‐negative microorganisms, along with viruses inhabiting the urinary system, are also readily detectable in urine and can be indicative of metabolic or pathological microbial activity (Kang et al., 2013; Lee et al., 2017; Yoo et al., 2016).

### 2.2 Molecular composition of uEVs

Urinary EVs contain proteins, nucleic acids, lipids and metabolites. In recent years, the Vesiclepedia repository (V4.1; microvesicles.org, accessed 17 July 2020) (Kalra et al., 2012) has expanded exponentially and, at time of this writing, contains data from 1254 EV studies, including 38,146 RNA entries, 349,988 protein entries and 639 lipid/metabolite entries. From this list, 89 studies (7%) used urine as EV sample source.

The protein composition of EVs pelleted at 100,000–200,000 × *g* from urine of healthy individuals has been extensively investigated. In these conditions, approximately 0.6–3% of the **protein** in urine is associated with this EV fraction (Bryzgunova et al., 2016; Zhou et al., 2006). The first mass spectrometry study of uEVs in 2004 (200,000 × *g* pellet) detected 295 proteins (Gonzales et al., 2009; Pisitkun et al., 2004). By 2009 the number of identified proteins reached 1132 (Gonzales et al., 2009; Pisitkun et al., 2004), likely due to improvements in mass spectrometric techniques. The use of newer generation mass spectrometry instruments has expanded the uEV proteome to over 3000 proteins, enabling deeper analysis of EV biology and identification of additional biomarker candidates (Bijnsdorp et al., 2017; Dhondt et al., 2020; Fujita et al., 2017; Stokman et al., 2019). Proteins identified in uEVs include membrane trafficking components, cytoskeletal proteins, motor proteins, membrane transporters, and glycosylphosphatidylinositol‐linked proteins (Zhou et al., 2006). In agreement with the idea of uEVs having diverse cellular origins, characteristic proteins of the different organs of the urogenital system, that is, the kidneys (glomeruli, proximal tubule, and distal tubule), the bladder and the prostate, have been detected in uEVs (Gonzales et al., 2009; Pisitkun et al., 2004; Street et al., 2017) (Table 1 and Figure 2). For comprehensive discussions of the proteomic analysis of uEVs we refer the reader to review papers on this topic (Erozenci et al., 2019; Merchant et al., 2017). The analysis of uEV surface markers by flow cytometry and Western blotting has confirmed the presence of uEVs derived from the cells lining all nephron segments (Table 1) (Gamez‐Valero et al., 2015). The presence of podocin, podocalyxin or nephrin indicate uEVs from glomerular podocytes, whereas the presence of megalin, cubilin, aminopeptidase or aquaporin‐1 (AQP1) indicate uEVs from proximal tubular cells. Uromodulin (UMOD, also known as Tamm‐Horsfall protein (THP), CD9, and type 2 Na‐K‐2Cl cotransporter (NKCC2) mark uEVs from the cells of Henle's loop and aquaporin‐2 (AQP2) marks uEVs from collecting ducts. CD133 identifies uEVs from proliferating/progenitor tubular cells (Dimuccio et al., 2014). Finally, bladder derived uEVs contain uroplakin (Pisitkun et al., 2004).

**Lipids and different metabolites** are also components of uEVs, but only a few studies have focused on these molecules (Clos‐Garcia et al., 2018; Del Boccio et al., 2012; Skotland et al., 2017). A recent lipidomic study identified over 100 lipid species by mass spectrometry in uEVs (100,000 × *g* pellet). These EVs showed a remarkably high content of cholesterol (63%), with phosphatidyl serine 18:0/18:1 being the next most abundant lipid species (Skotland et al., 2017). In addition, uEVs have a higher cholesterol content compared to plasma derived EVs (Skotland et al., 2019). Another recent study using targeted ultra‐performance liquid chromatography‐tandem mass spectrometry identified metabolites from five main categories of metabolites in uEVs (organic acids and their derivatives, nucleotides, sugars and derivatives, carnitines, vitamin B/related metabolites, and amines). The most abundant metabolites detected were ornithine, creatinine, D‐ribose 5‐phosphate, L‐cystathionine, alanine, and serine (Puhka et al., 2017).

The membrane of uEVs is highly decorated with a variety of **glycans** linked directly to the proteins and lipids of the EV membrane. The abundance of different glycosylations adds to the biomolecular complexity of uEVs and it has been shown that these integral structural and functional components play a role in EV uptake (Williams et al., 2019). Analysis of uEV carbohydrate content by mass spectrometry and lectin arrays demonstrated that uEVs are highly enriched in complex type N‐glycans, with terminal modification consisting of mannose and fucose residues (Kalluri & LeBleu, 2020; Saraswat et al., 2015). For a detailed review of EV glycosylation see Williams *et al*. (Williams et al., 2018).

The presence of **RNA** in EVs was discovered in 2006 and the first reports of mRNAs and miRNAs in uEVs followed soon after (Baj‐Krzyworzeka et al., 2006; Bryant et al., 2012; Miranda et al., 2010; Nilsson et al., 2009; Palanisamy et al., 2010; Ratajczak et al., 2006; Valadi et al., 2007). So far, most studies of small noncoding RNAs in uEVs have focused on miRNAs, but other noncoding RNAs such as small nuclear RNAs, small nucleolar RNAs, tRNAs and lncRNAs or fragments thereof have also been found in pelleted uEVs (Barutta et al., 2013; Cheng et al., 2014; Delic et al., 2016; Ghai et al., 2018; Srinivasan et al., 2019) and in SEC‐enriched uEVs (Lozano‐Ramos et al., 2018). Non‐coding RNAs were found to be the predominant nucleic acid cargo in the deep sequencing study of uEVs by Miranda *et al*. (Miranda et al., 2014). However, more than 13,000 protein coding genes were detected as well, along with abundantly present rRNA transcripts. A total RNA sequencing approach by Everaert *et al*. and a poly‐A based RNA sequencing approach targeting mRNAs by Barreiro *et al*., confirmed this vast representation, as they reproducibly detected transcripts from over 10,000 genes in uEVs, which was found to be the highest number of all evaluated biofluids (Barreiro et al., 2020; Everaert et al., 2019). Interestingly, uEVs were also shown to be a good source of novel RNA species, such as circular RNAs. In conclusion, many studies have shown the association of RNA and uEVs. However, since RNA can also be found in other molecular structures than EVs, it is recommended to show that the EV‐RNAs resist mild degradation by proteinases and nucleases (Mateescu et al., 2017; Thery et al., 2018; Veziroglu & Mias, 2020). It is not yet clear whether **DNA** is present in the lumen of uEVs, but DNA may be found on their exterior (Bryzgunova et al., 2016; Miranda et al., 2010). Concerning DNA in the uEV lumen, a study showed that no large differences were observed when comparing the read distribution of the uEV inner nucleic acid cargo with and without DNase I digestion following deep sequencing (Miranda et al., 2014).

These and many other studies have given us an overview of the molecular composition of uEVs. Nevertheless, it is generally recognized that the different EV isolation methods do not entirely remove all non‐vesicular material and that the methods separate distinct EV populations to a different extent (Thery et al., 2018). Hence when reviewing such data, care and caution are needed, as some of the identified molecules may not represent genuine EV‐related components and/or the specific EV population that is being investigated.

### 2.3 Physiological functions of uEVs

Increasing evidence indicates that EVs released into the urine can be internalized by other cells and can modulate their function, suggesting the presence of intra‐nephron communication along the urinary lumen (Gildea et al., 2014). By electron microscopy studies, EVs were shown to be internalized by proximal tubular epithelial cells through cilia in vitro (Hogan et al., 2009). Moreover, other in vitro studies showed that collecting duct‐derived EVs could be internalized by tubular cells, transferring AQP2 (Street et al., 2011). Treatment of cultured tubular epithelial cells with podocyte‐derived EVs induced a profibrotic phenotype, potentially identifying a novel form of glomerular‐tubular communication (Munkonda et al., 2018). Studies have also identified a role for uEVs in innate immunity (Hiemstra et al., 2014).

In addition, the accumulation of a diverse mixture of uEVs in the bladder followed by their expulsion from the body through urination strongly suggests a principal role for uEVs as a route of elimination. It remains undetermined if excretion through urine is the primary mode for eliminating EVs in general, including circulating ones, or whether mostly EVs from the genitourinary system are excreted in urine. The study of the physiological functions of uEVs is still in its infancy.

## 3 CURRENT STATE OF THE ART OF URINARY EV RESEARCH

### 3.1 Collection, processing, and storage of urine for uEV research

Urine collection, processing and storage are important topics that should be carefully considered in uEV studies because they are major sources of data variability and can limit reproducibility (Clayton et al., 2019; Dhondt et al., 2018; Zhou et al., 2006). Currently, only general guidelines like the Biospecimen Reporting for Improved Study Quality (BRISQ), including urinalysis and standards (ISO 20387:2018) are established for best practices in urine biobanking (Moore et al., 2011; Rabinovitch et al., 2009). Studies addressing collection, processing and storage of urine specifically for uEV research are very limited. The data can be profoundly influenced by the up‐front pre‐analytical variables, where biospecimen handling is subject to different methods, for example, in collection times, preservatives or centrifugation (Table 3). These differences can lead to selective and variable inclusion of EV subpopulations and non‐EV contaminants such as cells or their fragments, uromodulin networks and protein aggregates. Therefore, it is of utmost importance that the modality of urine handling is consistent within any study. In addition, for interoperability, it is essential that the reporting of such methods is also harmonized across research teams. The EV field would highly benefit from proficiency testing trials that could ideally be conducted in collaboration with biobanks (e.g., www.ibbl.lu/ibbl‐bioservices/biospecimen‐proficiency‐testing/). Within ongoing and future urine biobanking studies, we consider that special focus should be put on method validation/consistency and particularly on identifying the most and the least variable preanalytical parameters that affect EV research (Table 2).

**TABLE 2:** Reporting on urine collection, processing and storage

Parameters	Reporting priority level	Evidence level	What to report	Recommendation
Research subject information (demographical and clinical data)				
Species	Obligatory	High: There are clear species‐specific differences that impact all of their characteristics	Species, subspecies	Record: Species and subspecies information
Gender/Biological sex	Obligatory	High: There are clear gender/sex differences between urine biomarkers (e.g., creatinine, prostate EVs))	Male, Female, Genderqueer	Make sure to gender‐balance cohorts to be compared
Age	High/Obligatory	Medium: Based on mesenchymal stem cells and blood EVs (reviewed in (Boulestreau et al., 2020))	Age in years	Make sure to age‐match cohorts to be compared
Clinical Data, for example, diseases, kidney function parameters, medication, comorbidities	High/Obligatory	High: Clinical parameters are essential for disease/condition/organ‐related EV research	Clinical parameters in standard units	‐ Utilize urine dipstick ‐ Measure urine creatinine ‐ Measure disease‐specific markers (e.g., urinary PSA for prostate and albumin for kidney research) ‐ Record all relevant clinical parameters
Supporting information, for example, BMI, ethnicity, diet, fluid intake, geographical information.	Medium	Medium‐High: Certain supportive information is important to record as it might influence urine EVs	Supporting parameters in standard units	Determine relevant supporting information and record them: Based on the study goal, supporting information can be crucial
Urine collection				
Pretreatment	Obligatory	High: The most common pretreatment methods prior to urine collection (DRE, prostate massage, catheterization) can have an effect on the EV content of the sample(Duijvesz et al., 2015)	DRE and/or prostate massage (yes/no, Number of strokes) Catheterization (yes/no)	Any manipulation which could affect the composition of the urine should be reported in detail
Ethical approvals	Obligatory	N/A	Approving authority, Informed consent forms, collection details (origin, type and number of samples)	All collected samples should be linked to designated ethical approval, applied for uEV research
Collection method	Obligatory	Medium: The information of the transition of urine through the urethra is important particularly for disease‐related uEV studies	‐ Clean‐catch ‐ Sterile urine bag ‐ Assisted (urethral catheterization, suprapubic aspiration, pediatric specimen ‐ Animal collection cage	Details of the collection method for example, use of syringe, possible transfer of the sample to container
Time and type	Obligatory	Medium: uEV concentration can vary depending on the urine transition time from the bladder	‐ Collection type (morning/random/spot) ‐ Timed collection, for example, 24 h	Type of collection for example, random/spot urine, first or second morning urine. Record: Time between the last uncollected and collected void
Volume and void	Obligatory	Medium: The collection of first void urine transitioning from the urethra may affect the uEV quantity/composition	‐ Void (first/mid/full) ‐ Volume in ml	Collection of midstream urine is recommended to avoid microbial contamination
Collection device and container type	Medium	High‐Medium: Certain containers and devices may have an effect on the uEV content; for example, the material may bind EVs or contain microbial contaminants if not sterile	‐ Brand ‐ Sterile yes/no ‐ Material ‐ Open/closed	The container should be clean, leak‐proof, urine pH‐range resistant and not shed plastic particles. Record: Material, manufacturer, lot number
Storage prior to processing				
Storage time	Obligatory	High: Longer storage time may lead to microbial growth, cell debris and particularly to degradation of more labile biomolecules (e.g., RNA)	Hours	Samples should be stored max. 8 h before processing
Storage temperature	Obligatory	High: Freshly collected urine samples should be cooled promptly to avoid microbial growth or biomolecule degradation	Degrees Celsius	Max 4°C is recommended
Light protection	Medium	Low: Some urinary analytes may be light sensitive (e.g., bilirubin, porphyrins); impact on uEVs unknown	Light protection (yes/no)	Use of amber‐coloured/dark collection tubes
Urine quality control				
Use of dipstick	High	High: Presence of for example, cells, microbes and high protein levels affects purity and composition of uEV population	‐ Yes/no ‐ Brand ‐ Deviating parameter(s)	Recommended for preliminary urine assessment (pH, protein level) and exclusion of deviating samples (blood, microbes)
Preprocessing				
Collection container preparation	Medium	Medium: Preservative might be affected by time and storage in collection container	‐ Preservative already present in collection container (yes/no) ‐ Preservative in container freshly prepared (yes/no)	‐ Keep the protease inhibitor cocktail on ice or at the manufacturer's recommended temperature at all times ‐ If protease inhibitors are used at collection time, it is recommended that sample containers are prepared by adding protease inhibitor cocktail and keep frozen at ‐20°C for max. 6 months until use ‐Alternatively, prepare fresh and use immediately
Urine sample preprocessing	High	High: Freshly collected urine samples should be cooled promptly to avoid microbial growth or biomolecule degradation	‐ Time ‐ Temperature	‐Process urine within 4–6 h from sample collection ‐Consider addition of protease inhibitors or preservatives when fast processing (>6 h at 4°C) is not possible (see below)
Urine centrifugation	Obligatory	Medium: 800 xg to sediment cells and debris without damaging urine cells	‐ G‐force ‐ Volume/tubes ‐ Temperature ‐ Time	‐ Homogenize urine sample before centrifugation ‐ G‐force range 500 to 800 g ‐ Centrifugation at 4°C
Recovered supernatant (method/volume)	High	Medium: Largely operator‐dependent	‐ Pipetting, decanting, pouring ‐ Recovered volume	‐ Loose pellets (low speed centrifugation, e.g. <1000 × *g*): Pipetting without disturbing the pellet is recommended to avoid pellet carry over ‐ Tight pellets: uniform procedure for all samples
Other urine fractions	Low	Medium‐High: To monitor the purification process of EVs	‐ Pellet ‐ Whole Urine	‐ Less‐used source of EVs ‐ Collection for use as controls or exploration of EVs in these fractions is recommended
Collected aliquots of supernatant	Obligatory	Medium: As samples may be used for several techniques/isolation protocols, aliquots of different volume may be required to avoid repeated freeze/thawing and to optimize workflows and storage capacity	‐ Number of aliquots ‐ Date ‐ Volume (if different volumes are collected)	‐ Immediate freezing at ‐70°C or colder is recommended after aliquoting ‐ Suggested volumes of aliquots: Large (up to 30 ml) Medium (5–10 ml) Small (1–2 ml)
Storage				
Storage container	High	Medium: Should resist pH range of urine and not shed any particles, low EV (protein or lipid) binding properties generally beneficial	‐ Brand ‐ Volume	Use of ¾ of the maximum volume of the container is recommended to accommodate the expansion of the sample due to freezing
Temperature	Obligatory	Medium‐High: EV yield may be lower from samples stored at ‐20°C	‐Degrees Celsius	‐70°C or colder is recommended
Method of freezing	High	Low: Quick freezing is generally recommended to preserve biological specimens, but tests and impact on uEVs of about speed of freezing speed or cryoprotective agents in urine are lacking	‐ Snap freezing in liquid nitrogen ‐ Freezing at a freezer ‐ other if applicable, for example, gradual freezing or use of cryoprotective agents	Freezing quickly at ‐70°C or colder or in liquid nitrogen is recommended
Defrosting				
Temperature	Obligatory	Low: The effect of thawing temperature on uEVs has not been extensively studied, but might affect heat labile biomolecules or to sediment formation	Degrees Celsius	‐ Record: The temperature(s) at which the sample has been thawed
Method	Obligatory	N/A	Heating pad, water bath, incubator, room temperature, refrigerator	‐ If applicable, the model and type of the device used for the thawing ‐ Defrosting should be done equally for all compared samples
Time	High	Medium: For longer thawing times preservatives may be needed	Minutes, hours	‐ Record: The time it takes to completely thaw the sample ‐ Prolonged warming not recommended to avoid microbial growth
Additives at time of collection:				
‐ Protease inhibitors ‐ RNase Inhibitors ‐ Chemical preservatives, for example, azide	Obligatory	Medium: Preservatives inhibit microbial growth and protease inhibitors preserve certain urine proteins (many proteins are not prone to proteolysis)	‐ Type ‐ Name ‐ Brand ‐ Final concentration ‐ Stage/time at which additive was used (to whole or pre‐cleared urine)	‐ Relevant only for longer collection times (inhibiting microbial growth) or for specific down‐stream EV applications (e.g., surface antigen characterization). ‐ Preferably use preservatives targeting specific enzymes (e.g., RNase), as general (RNA) protecting agents likely affect EVs ‐ Add selected preservatives immediately at the time of urine collection
Sample transportation				
Temperature	Obligatory	Medium‐High: EV quality and quantity diminish with long‐term RT and by multiple freeze‐thawing. Preservatives can prevent protein/RNA breakdown and bacterial outgrowth	‐ Degrees Celsius at transport and degrees Celsius at arrival ‐ Cooling system, when applicable (e.g., ice)	Aliquot urine and freeze at ‐80°C to be transported frozen at ‐80°C. For non‐aliquoted fresh urine (e.g., home‐testing), immediate transport at RT or 4°C can be considered, particularly when preservatives are added
Time and method	High	Medium‐High: EV quality and quantity diminish with long‐term at RT. Container leakage could introduce contamination	‐ Transport duration in hours ‐ Container damage/leakage	Record: Transport duration and container damage
Existing biobanks				
Existing urine sample collections	N/A	High: Existing urine biobanks with protocols not optimal for EV preservation are often used for research	N/A	‐ Collect all above‐mentioned parameters and determine appropriateness of the sample collection for your research purpose ‐ Perform tests to determine urine quality, number and characteristics of EVs as described in sections 3.3‐3.4

Reporting Priority Level is primarily meant to indicate the importance of recording a specific parameter in a biobank database. Not all of these parameters are relevant for publication reports. The Evidence Level is an expert consensus opinion of the current level of confidence that the parameter is a variable to consider during sample biobanking and data analysis and interpretation.

Individual research studies have typically employed different urine collection and storage approaches. This is often a result of study‐specific protocols and/or logistic restrictions. Large professional biobanks are designed to allow measurement of a wide variety of urine analysis parameters, meaning that the sample collection and storage protocols used might be sub‐optimal for uEVs. Therefore, it is unlikely that a universal pre‐analytical procedure will be adopted for all uEV studies. Instead, it is more likely that different best practice protocols will be established depending on the molecular component of interest, the choice of analytical platform(s) and the investigated health condition or disorder. As long as standard operating procedures for collection and storage of uEVs are not established by the community, it is safest to report all available pre‐analytical information related to the studies in the EV‐TRACK knowledgebase, in accordance with the Minimal Information for Studies of Extracellular Vesicles 2018 (MISEV2018) and other ISEV rigor initiatives as well as other suited guidelines developed particularly for preanalytical variables of fluid samples (Betsou et al., 2010; EV‐TRACK Consortium et al., 2017; Lehmann et al., 2012; Lotvall et al., 2014; Nanni et al., 2012; Thery et al., 2018; Witwer et al., 2013). This will enable a better understanding of the impact of these variables and ideally enable more meaningful comparisons between different studies. In the future, the evaluation of pre‐analytical conditions could be used to establish case‐specific “Best Practice” protocols. Below we provide the current state of the art of uEV research which also includes common practices. This will be followed by consensus recommendations and an indication of knowledge gaps in the field of uEV research.

#### 3.1.1 Patient information

Demographic and clinical parameters including gender, age, ethnic background, weight, height, fluid intake, diet, time of urine collection, laboratory measurements and medication and so forth should be recorded to identify potential sources of variability, confounders and introduction of unintended bias through the selection of inappropriate members in these cohorts (Ransohoff & Gourlay, 2010). When possible, particular attention should be paid to clinical information about kidney function (e.g., glomerular filtration rate, albuminuria) as a pathological condition of the kidney has a major effect on the urine and uEV composition (Simeone et al., 2020). Kidney pathology may also affect uEV excretion, potentially biasing normalization at a later stage (see below). A good example of a study in which careful clinical characterization was done and kidney disease was ruled out as a confounder is a recent examination of uEV cargo as markers for neurological disorders (Wang et al., 2019). It is also important to record a patient's use of diuretics or other drugs which may drastically affect urine composition and pH. pH has been reported to affect uEV physiology and isolation (Parolini et al., 2009; Zhao et al., 2017). In addition, urinary pH is highly influenced by diet, that is, vegetarian diet causes a high alkaline load (Trilok & Draper, 1989; Trilok & Draper, 1989). Therefore, reporting general dietary information may improve interpretation of results. Guidelines for appropriate biospecimen reporting for initiation of studies have been developed by several organizations, and some offer online tools to assist with this (Cheah et al., 2012). Nonetheless, detailed information about the patient population under investigation is an aspect that is notoriously under‐reported in the literature, a recognized general failing of biomarker studies (Moore et al., 2011).

#### 3.1.2 Urine collection types and variables

##### Instructions and donors

Urine collection is typically performed by the donors themselves. Thus, before the collection, clear and concise instructions on the sample collection process including appropriate hygiene should be given, ideally in both spoken and written forms. As the collection methods may be quite complex or laborious and instructions as well as donors differ greatly, highly standardized collections are difficult to achieve (Fisher et al., 1977).

##### Time and void

Urine can be collected during a single voiding episode (“spot urine collection”) or can be collected across several voiding episodes during a fixed time period (“timed urine collection”). Spot urine collections can be done at a random time (“random” spot urine) or standardized to the first or second morning urine. Timed urine collections can be over the course of hours or a day (called “24‐hour urine”). The volume of urine collection can be “full void” or “midstream urine” (e.g., without collecting the earliest portion of the voided urine). Relatively little is known about the impact of different collection types on uEV measurements.

The first morning urine is generally more concentrated than a random spot urine (Thomas et al., 2010), possibly resulting in a higher uEV concentration in the first morning urine. Zhou *et al*. found only minor differences between first and second morning urine with respect to total protein in uEVs or exosome‐associated proteins (Zhou et al., 2006). Another study of uEVs from first and second morning voids in three control males showed that only 4% of the identified proteins by mass spectrometry were significantly altered in abundance between the two conditions (Øverbye et al., 2015). Nevertheless, specific uEV biomarkers may fall within this fraction, and it is therefore recommended to determine the stability of identified biomarkers in relation to pre‐analytical variables. In addition, physiological processes in the kidney and some kidney bio‐markers follow a circadian rhythm (Firsov & Bonny, 2018). It is currently unknown whether the release of uEVs or the composition of their cargo demonstrate a circadian rhythm in humans, although one study has examined these questions in rodents (Koritzinsky et al., 2019). Periodicity would be discovered only by analysing timed urine collections, ideally gathered in fractions over 24 hour (Firsov & Bonny, 2018).

In the case of a timed collection, documenting and reporting the time between the last uncollected and first collected void would help with assessing urine transition time in the bladder and may be of additional value for normalization. For example, uEV protein content could be related to a time period of 4 or 6 h, which might be easier to collect than 24‐hour urines. In many prostate cancer studies, urine samples are collected after a digital rectal examination (DRE) by the urologist. Collection at this time point can greatly increase the amount of prostatic fluid in the urine and consequently enriches the sample for prostate‐derived EVs (Duijvesz et al., 2015; Fujita & Nonomura, 2018; Hendriks et al., 2016).

The collected urine void impacts the availability or enrichment of specific EVs and other urine components. First void after DRE has been shown to increase the chance of finding prostate cancer associated EVs (Fujita & Nonomura, 2018; McKiernan et al., 2016). However, first void also contains more cells and bacteria than the mid‐stream void, leading to 36% of urine samples to exceed health related upper reference limit versus 10% of mid voids (Manoni et al., 2011). It is unclear which urine collection is the “cleanest” without significant contamination by cells or bacteria. Reduction of microbe content requires attention to the entire uEV workflow (Tataruch‐Weinert et al., 2016). Another point to be addressed is the need to establish an optimal workflow that addresses the presence of bacterial outer membrane vesicles (OMVs) in urine (derived from either normal or pathogenic urinary tract microbiota) (Barreiro & Holthofer, 2017; Lee et al., 2017; Yoo et al., 2016). Another mechanism that may influence EV secretion rate includes urinary flow; that is, kidney tubule cells have cilia that may be activated by flow and have an important role in EV secretion (Wang & Barr, 2016). However, the in vivo implications have not been studied.

Little is known about the inter‐day variation of uEVs. For example, Wang and others investigated the variability of the uEV proteome in morning urine from two healthy volunteers over a two‐month period (Oeyen et al., 2019; Wang et al., 2019). They showed that approximately 50% or hundreds of uEV proteins were stable at the inter‐day and intra‐individual level. As expected, most variation was found within the low abundance proteins. Some of the stable proteins could be classified as housekeeping, including numerous heat shock proteins, actin and annexin A4. On the RNA level, Murakami *et al*. (Murakami et al., 2014) have found that the expression of some uEV mRNAs from different parts of the kidney were stable on the intra‐individual level over a two‐week period. On the other hand, larger inter‐individual differences were found. While the authors could confirm the stability of five mRNAs among the subjects, further studies are needed for discovery and validation of truly stable control uEV RNAs.

##### Collection containers and devices

Urine collection containers are typically made of plastics, such as high‐density polyethylene or polypropylene, can be sterile or unsterile, open or closed, anatomically compatible or have tube transfer systems. Some even have a urine temperature thermometer affixed to the outside of the cup. There are no studies known to have tested the impact of different containers on uEV collection. However, it is important to ascertain that containers should not bind uEVs or shed (plastic micro‐) particles. Models with a lid are preferable to prevent the introduction of external EVs. Sterile tubes may be especially important for studying microbial uEVs. Specialized collection devices might be needed (e.g., urine bag for infants) or part of a protocol for standardized collection of different voids (e.g., first 20 ml void using a Colli‐Pee device, Novosanis, Belgium).

##### Preservation: storage before freezing

Unprocessed urine should be kept at 0–4°C and processed within to 8 h to avoid bacterial growth, cell lysis, molecular degradation of RNA and protein, and formation of sediments (Barreiro et al., 2020; Moatamed, 2019; Saetun et al., 2009). However, it may not be universally recommended to keep urine cold. Armstrong *et al*. found that miRNA and other small RNA contents of uEVs declined during 4–24 h of storage after collection, and the decline was greater when samples were kept at 2–4°C rather than at room temperature (RT) (Armstrong et al., 2018). The authors discussed that the decline could be due to cold induced precipitation and that it could be rescued by warming the urine sample for 5 min at 37°C. Indeed, heating increased RNA yields from frozen samples that had formed precipitates. However, this could also be related to the formation of uromodulin polymers that form when urine is kept cold, for example, below 4°C (Wachalska et al., 2016). These polymers can trap EVs to some extent, which are subsequently removed from the sample after low‐speed centrifugation (Wachalska et al., 2016).

With longer timed collections, such as 24 h collections, fast processing cannot be achieved, and studies of the possible effect of repeated warming (37°C) and cooling (either to RT or +4°C) of the urine specimen during collection are lacking. However, generally, if long urine collection times are required, the addition of preservatives such as azide should be considered to avoid microbial overgrowth, at least when the preservative is compatible with further uEV processing steps (Havanapan & Thongboonkerd, 2009; Thongboonkerd & Saetun, 2007). Effects of RNase inhibitor addition have not been investigated systematically, even though RNAses are present in urine.

Several studies have investigated whether protease inhibitors should be added to urine to avoid uEV protein degradation (Mitchell et al., 2009; Zhou et al., 2006). Although this may preserve some specific uEV proteins such as NKCC2, analysis of CD9 and TSG101 showed that not all EV proteins are prone to proteolysis in urine (Mitchell et al., 2009; Zhou et al., 2006). It is important to address this issue more conclusively because urine samples in biobanks are not typically collected with protease inhibitors because the use of protease inhibitors would increase costs considerably, especially in large sample studies. Similarly, when analysing phosphorylated proteins, the use of phosphatase inhibitors should be considered although it has not been thoroughly studied.

##### Urine quality control

Commercially available dipsticks can be used as a form of rapid quality control by measuring urine pH and various contents (e.g., leukocytes, erythrocytes, protein, glucose, nitrate, ketones, blood, bilirubin, urobilinogen) (Øverbye et al., 2015; Royo et al., 2016; Welton et al., 2010). Information obtained by these rapid, simple procedures identify patient status and allow exclusion of deviating samples, such as those heavily contaminated by microbial infection or blood. However, dipstick use for inclusion/exclusion in uEV studies has been rather arbitrary to date: there is no consensus in defining which dipstick test is most suitable, or on where to set inclusion/exclusion criteria.

##### Clearing before freezing

Frozen, precleared urine is used in many uEV studies. Preclearing usually involves centrifugation to remove cells, large cell debris and often also the bulk of uromodulin, and it is done so that these materials do not contaminate uEV preparations with artifactual, similarly sized particles during freeze‐thaw cycles. Interestingly, however, one study found that uEV miRNA/small RNA correlated highly when comparing urine aliquots that had been centrifuged alternatively before freezing or after a freeze‐thaw (Armstrong et al., 2018).

Several urine processing protocols are currently available from uEV researchers and biobanks for example, the European Association of Urology Standard Operating Procedures in UroWeb (uroweb.org/research/how‐we‐work/) and the MMI Guidelines for Standardized Biobanking (crdi.ie/resources/biobanking‐guidelines/) from the Clinical Research Development Ireland. According to these sources as well as other uEV literature, centrifugation parameters used for preclearing vary widely. For example, the centrifugation speed used ranges from roughly 1–20,000 × *g*, centrifugation time varies between 0–30 min, and both one‐ and two‐spin approaches are used. Centrifugation volumes, use of a brake and supernatant removal methods also vary between studies, although these parameters are rarely specified in publications. Low speed centrifugation (<1000 × *g*) is generally used to remove whole cells and large cell debris, but data has shown that lower speeds may not suffice for this task. A single 400 × *g* step for 5 min results in inadequate removal of cells while efficient cell pelleting was achieved by centrifugation of 10 ml volumes at 1358 × *g* for 10 min in round bottom tubes (Bunjevac et al., 2018).

Uromodulin, also known as Tamm‐Horsfall protein, is the most abundant protein excreted into urine (Micanovic et al., 2018). Most uromodulin can be sedimented with a 2000 × *g* spin for 30 min without a gross loss of uEVs, whereas speeds ≥ 10,000 × *g* result in pelleting of uromodulin and EVs (Fernandez‐Llama et al., 2010; Musante et al., 2014; Musante et al., 2020; Puhka et al., 2017). Some studies have combined first a lower speed spin, for example, 300 × *g*, with a second higher speed spin, for example, 2000 × *g* (Mussack et al., 2019), to deplete the larger and smaller contaminants consecutively. While this method might be more effective than a single spin, it also adds to the handling time and steps, which can be limiting for large sample numbers. Loss of EVs due to binding to polymeric uromodulin can be reduced or eliminated through use of reducing agents that depolymerize uromodulin by breaking disulfide bridges between individual uromodulin monomers (Fernandez‐Llama et al., 2010).

The choice of preclearing parameters usually depends on the study goal. Ammerlaan *et al*. optimized urine processing (centrifugation speed, time, temperature and brake) for reproducibility in proteomic and metabolomics studies with the criteria that the urine supernatant should still contain the EV component (Ammerlaan et al., 2014). As depletion of larger EVs (microparticles) was preferred in the original study, the best protocol with low microparticle counts in the recovered supernatant was a 20 min, 12,000 × *g* centrifugation at 4°C with a hard brake. An optimal pre‐clearing/pre‐freezing protocol might thus be EV subtype‐specific, but in practice, compromises may be necessary when using biobanked samples, because these resources are designed to provide urine samples for a variety of uses.

##### Collection volume and freezing aliquots

The volume of urine required for uEV analysis depends on the yield of the method used to isolate EVs and the sensitivity of the analytical method, but 10–30 ml of urine is sufficient for many purposes, for example, RNA sequencing or proteomics (Musante et al., 2020). It is advisable to collect and store processed urine in aliquots (as a backup or for use in different analyses). Most urine collection containers collect sufficient volume to allow division into multiple aliquots of suitable size (e.g., 1 to 30 ml) which speeds up the freeze and thaw processes and avoids unnecessary pooling of aliquots or multiple freeze/thaw cycles. Whenever possible, it is recommended to preserve the cellular pellet or the low‐speed centrifugation pellet, which contains also uEVs (Musante et al., 2020), as well as aliquots of whole urine for monitoring of the purification process, for comparative analyses or as controls.

##### Freezing temperature and storage time

Freezing and storage at ‐70°C or lower temperature is preferred. Zhou *et al*. showed that storage at ‐20°C caused more than 50 % loss of EVs compared with storage at ‐80°C where EV loss was 14 % (Zhou et al., 2006). Partially supporting these findings, Oosthuyzen *et al*. measured particle count by nanoparticle tracking analysis (NTA) in urine samples stored at room temperature, at +4°C, or frozen for 2 h to 1 week (Oosthuyzen et al., 2013). Particle counts were lower in the samples stored at ‐20°C compared with ‐80°C or other temperatures. Protease inhibitors in this study also had a positive effect increasing the recovery of particles from less than 40% to over 80% from the original counts. A later study reported that concentration and particle size remain similar after freezing at different temperatures, that is, ‐20°C, ‐80°C or ‐196°C without gross changes in uEVs morphology as observed by TEM. Particle concentration analysis by NTA showed approximately 2‐fold increase and similar decrease as measured by resistive pulse sensing (RPS), in comparison with fresh samples. (Yuana et al., 2015). Particle mode size increased by 17% during 1 year of storage at ‐80°C. Overall, uEVs were found to be more stable during 1‐year storage at ‐80°C as compared with EVs from other body fluids. Thus, most evidence for uEVs storage temperature is in line with the recommendations for EVs from other body fluids and storage at ‐70°C or colder is recommended (Cheng et al., 2019). Regarding antigenicity after freezing and long‐term storage, it is of importance to note that the uEVs proteome includes thousands of proteins, therefore it cannot be excluded that some proteins can be more prone than others to lose antigenicity after long term storage. For example, in The Finnish Diabetic Nephropathy Study (www.finndiane.fi), a well‐established cohort of urine samples with different levels of albuminuria, many isolated uEVs were associated with antigens including proteases, protease inhibitors and ubiquitin (Musante et al., 2015). These proteins might lead to loss of antigenicity in different cohorts after thawing, but the process of freezing and thawing by itself could affect the antigenicity. Only dedicated studies of uEVs can established the best condition for freezing temperature and storage time for the protein(s) under investigation.

Frozen uEVs might interact with cryoprecipitates, mainly calcium oxalate dehydrate and amorphous calcium or polymerized proteins, leading to uEVs entrapment and apparent loss unless released by measures such as vortexing, dilution, lowering of ionic strength or depolymerization of proteins (Puhka et al., 2017; Saetun et al., 2009). Early studies reported that vortexing after thawing can considerably increase uEV recovery from urine frozen either at ‐20°C (87% recovery) or ‐80°C (to 100% recovery), even after 7 months of storage (Zhou et al., 2006). However, it is not known if vortexing damages vesicles or if it leads to loss of luminal content and other studies did not observe significant effect of post‐thaw vortexing (Oosthuyzen et al., 2013). Additional work is needed to investigate in a comprehensive manner to which extend the size, number and molecular composition of uEVs is affected by freezing temperature and storage time.

### 3.2 uEV separation

Several EV separation methods that show specific advantages and disadvantages have been developed (Coumans et al., 2017; Konoshenko et al., 2018). Moreover, the selected isolation method may affect the characteristics and analysis of both isolated EVs and contaminants (Alvarez et al., 2012; Merchant et al., 2017; Mussack et al., 2019; Royo et al., 2016; Royo et al., 2016). A main focus has been on purity and yield of uEVs and usually one improves at the expense of the other. In addition to yield and purity, emphasis should also be given to practical considerations such as speed, scalability and throughput, as any high‐impact clinical research, and biomarker research in particular, requires validation of the results in hundreds to thousands of samples. Further, all isolation techniques yield only a subset of uEVs, which does not necessarily contain all uEVs of interest. However, in some cases, specific enrichment of a subset may be advantageous and improve the detection of some markers.

Traditionally, uEVs have been separated by ultracentrifugation. However, “ultracentrifugation” is not one technique, and there are a host of protocol variants and specifics across studies contributing to variable results within this category of separation modality (EV‐TRACK Consortium et al., 2017)). Sequential centrifugation is more commonly used and involves low speed centrifugation to remove cells and debris, followed by the subsequent consecutive collection of large and small EVs at increasing centrifugation speed (in general 10,000–20,000 × *g* for 20–30 min for large EVs, and 100,000–200,000 × *g* for 1–2 h for smaller EVs) (Gonzales et al., 2010). However, it has been reported that ultracentrifugation (UC) can have poor efficiency, with up to 40% of small uEVs retained in the supernatant after UC at 200,0000 × *g* (Musante et al., 2013).

A major challenge to effective EV separation is the highly abundant urinary protein uromodulin, which forms long polymers that can entrap small EVs (Musante et al., 2020; Pisitkun et al., 2004). Trapped EVs will then co‐pellet with uromodulin at low centrifugation speeds and may reduce the recovery of small uEVs isolated by sequential centrifugation (Figure 1a). Several approaches have been shown to release entrapped vesicles such as addition of the reducing agent dithiothreitol (DTT) Tris (2‐carboxyethyl) phosphine hydrochloride (TCEP‐HCl), the detergent 3‐[(3‐Cholamidopropyl) dimethylammonio]‐1‐propanesulfonate (CHAPS) or alkaline buffers) (Fernandez‐Llama et al., 2010; Musante et al., 2012; Musante et al., 2020; Puhka et al., 2017). Some groups have reported that DTT only slightly improves the yield (Cheng et al., 2014). A recent study demonstrated that removal of uromodulin using TCEP‐HCl does not affect particle counting with NTA/Tunable Resistive Pulse Sensing (NTA/TRPS), or results of flow cytometry or, qPCR, but did influence Western blotting and mass spectrometry results (Musante et al., 2020). Of note, detection of antigens depends on the analytical technique in use. SDS‐PAGE followed by Western blot is usually performed in reducing conditions without affecting the detection of the antigen of interest. However, there are exceptions depending on the type of antibody used and the nature of the antigen studied. For example, detection of tetraspanin by Western blot seems to be favoured when the sample is solubilized without any reducing antigen (Musante et al., 2017). Nevertheless, this evidence originates from very few examples and it would not be correct to extend this conclusion to the whole uEVs proteome which accounts for more than a thousand proteins. In addition, the use of reducing agents does not seems to affect the integrity of uEVs as reported by electron microscopy pictures of several studies which included either the use of DTT and / or TCEP (Fernandez‐Llama et al., 2010; Musante et al., 2020). A heterogeneous population of EVs was found in the pellet with size and morphology not dissimilar from the fraction before treatment including multi‐lamellar or composite structure with smaller EVs enclosed in larger ones. These findings reaffirm the importance of depleting uromodulin for certain downstream uEV analyses.

Disease‐related changes in urine content, such as proteinuria, can also complicate EV isolation. In particular, albumin (and other proteins) that leak into the urine in glomerular disease can bind to the surface of EVs or co‐elute as a protein complex (Merchant et al., 2017; Santucci et al., 2019). This can impair certain ultrafiltration‐based approaches and interfere with protein‐based characterization, for example, mass spectrometry or Western blot following ultracentrifugation (Rood et al., 2010). Coupling ultracentrifugation with size exclusion chromatography, the use of sucrose or other density gradients, or the isolation via filtration dialysis have been shown to reduce albumin and other proteins in EV isolates (Musante et al., 2014; Santucci et al., 2019). Another consideration is that proteinuria can alter urine viscosity which is a critical determinant of EV recovery in centrifugation‐based approaches (Inman et al., 2013; Momen‐Heravi et al., 2012). However, the impact of changes in urine viscosity in proteinuria on EV recovery is not known. The presence of red blood cells in urine samples (hematuria) can also alter the purity of EV isolates. A trypsin treatment performed before uEV isolation was recently described to prevent hematuria‐related proteomic alterations (Raimondo et al., 2018).

Many of the new methodologies developed for EV separation have been applied to uEVs, including filtration, precipitation, hydrostatic dialysis, ultrafiltration combined with size exclusion chromatography, acoustic trapping and immunocapture (Cheruvanky et al., 2007; EV‐TRACK Consortium et al., 2017; Dhondt et al., 2020; Huebner et al., 2015; Ku et al., 2018; Lozano‐Ramos et al., 2015; Merchant et al., 2017; Musante et al., 2014; Oeyen et al., 2018; Svenningsen et al., 2020; Wang & Sun, 2014). The efficacy and yield, and potential contaminants of these EV isolation techniques still need to be evaluated. Multiple studies have shown that the choice of isolation method can have a significant effect on measured EV molecular content (Freitas et al., 2019; Srinivasan et al., 2019). Co‐isolation of abundant proteins in urine with uEVs hampers the detection of less abundant proteins in uEVs. One strategy to account for high‐abundance uromodulin contamination in mass‐spectrometry proteomics analysis of uEVs is the use of an exclusion list of uromodulin peptides (Hiemstra et al., 2011). However, it should be noted that highly abundant proteins will still influence the identification and quantification of low abundant peptides, even with computational filtering after spectrum acquisition.

Finally, it is important to investigate to which extend the different EV isolation methods remove potential molecules/structures that may contaminate the uEV pellet. Main contaminants of uEV pellets can be bacteria, blood cells and lymphocytes, uromodulin and albumin. In normal conditions the main contaminant of the uEV fraction is considered to be uromodulin. Urine test strips can be used to detect abnormal levels of bacteria and protein (albumin), and the presence of blood cells and lymphocytes in the urine samples. A gel electrophoresis can also show the protein pattern of each urine sample before and after uEV enrichment. This information can be considered during sample inclusion as well as when analysing the uEV fraction. Electron microscopy can be used to detect the presence of abnormal vesicle morphology or other structures in the uEV sample, such as uromodulin (precipitates). Western blot can be used to detect specific co‐isolating proteins that may be present in the EV sample such as uromodulin or albumin. Proteomics analysis is also useful as it allows to compare the abundance of these and other co‐isolating proteins in relation to uEV proteins.

In conclusion, we note limited consistency and coherence between uEV separation methods. These limitations also apply to methods for characterization and analysis of EVs (addressed in the next section).

### 3.3 uEV characterization

#### 3.3.1 Post‐separation characterization and analysis of enriched uEVs

Authors reporting uEV characterization should be guided by MISEV reporting requirements (Thery et al., 2018). There are additional specific considerations for uEVs since urine is a particularly dynamic body fluid that contains EVs derived from a variety of cells. Parameters that vary in urine include concentration, osmolality, electrolytes, pH level, excreted/secreted proteins as well as cellular, bacterial, and viral quantity and content. There is no single technique that can characterize uEV heterogeneity by describing EV morphology, size, count and content. Each post‐isolation characterization is affected by the EV separation method used (see section 3.3.2 for further discussion of this topic). In many cases (i.e., animal work, archived random time spot urine) the amount of urine available may limit the number of complementary analyses that can be conducted (Musante et al., 2020).

The **morphology of uEVs** has been described by transmission electron microscopy (TEM), cryogenic electron microscopy (cryo‐EM), atomic force microscopy (AFM) and super resolution fluorescence microscopy (Figure 1). In particular, TEM and cryo‐EM show a heterogeneous group of EVs of different sizes and shapes, and cryo‐EM also allows the visualization of intraluminal structures (Musante et al., 2020) (Figure 1a). In addition to providing information about uEV size distribution, EM can also be used to assess sample purity, as gross protein aggregates, major vault proteins and other structural contaminants can be visualized and distinguished from EVs. See for example, the presence of uromodulin in uEV samples in Figure 1. Cryo‐EM preserves EV morphology and shows the lipid bilayers at high resolution, making it well suited for structural characterization of the EVs. However, performing systematic quantification of these parameters by cryo‐EM is time consuming and thus low throughput. Additionally, cryo‐EM requires costly equipment and specialized technical staff, which limits its accessibility and makes broader adoption of this approach to quality control unlikely. TEM (Figure 1b) also requires specialized facilities, but is generally more accessible. TEM negative staining protocols are straightforward, allowing visualization and sizing of EVs, and rough estimation of their purity in a large number of samples relatively quickly. TEM can also show EVs heterogeneity by differential staining densities to highlight morphological characteristics and surface features. Recently, super‐resolution microscopy has been used to directly visualize fluorescently labelled molecules within vesicles with 20 nm resolution, revealing the biomarker distribution and expression levels on single vesicles (Saliba et al., 2019). Many investigators have found SDS‐PAGE gel electrophoresis of the uEV sample to be useful as a general profiling tool to explore the protein pattern and detect potential protein degradation or protein contaminants, such as uromodulin (Musante et al., 2014; Musante et al., 2020; Zhou et al., 2006). Of note, uromodulin is rarely fully eliminated from uEV preparations, regardless of the separation method used, because there is a GPI‐anchored, membrane‐associated form of uromodulin which may be a normal constituent of tubular cell‐derived EVs (Musante et al., 2020; Rindler et al., 1990).

**uEV size distribution and counts** can be measured with commercially available particle analysers including NTA, based on Brownian motion and tunable resistive pulse sensing (TRPS), based on the Coulter principle (Musante et al., 2020; Oosthuyzen et al., 2013.). Both methods are discussed below, however there is only limited data comparing these methods.

Several technologies are utilized to study **EV content** (e.g., proteins, RNA, lipids, glycans). Western blot or ELISA techniques are based on bulk analysis of uEV content, whereas flow cytometry offers high‐throughput single‐EV surface protein analysis but requires advanced instrumental setup and experience to obtain sufficient resolution (Welsh et al., 2020). In addition, specialized cytometers with higher scatter sensitivity to measure small particles are not widely available (high‐resolution flow cytometry). As an alternative, bead‐based cytofluorimetric analysis can provide semi‐quantitative analyses of EV surface markers (Monguio‐Tortajada et al., 2019; Suarez et al., 2017). Recently, a bead‐based commercial kit detected up to 37 surface markers of EVs captured by CD63, CD9, and CD81‐coated beads (Wiklander et al., 2018). For uEVs, CD24, and CD133 might be of interest as markers of kidney function whereas other markers may identify kidney infiltrating cells (Dimuccio et al., 2020). Numerous omics analyses have also been performed to define the molecular content of uEVs and identify novel biomarkers for several diseases. Such studies include (small) RNA‐Seq and other transcriptomics analyses as well as mass spectrometry‐based proteomics (Carreras‐Planella et al., 2020; Carreras‐Planella et al., 2020; Cheng et al., 2014; Erozenci et al., 2019; Everaert et al., 2019; Park et al., 2020; Rodriguez et al., 2017; Salih et al., 2016; Srinivasan et al., 2019; Stokman et al., 2019; Thomas et al., 2016). Metabolomic and lipidomic studies of uEVs are also under development, but remain rather complicated, with workflows requiring specialized instrumentation and expertise (Clos‐Garcia et al., 2018; Williams et al., 2019).

Recently, a systematic comparison of 10 different isolation methods for small RNA EV‐cargo across 5 biofluids revealed marked differences in the complexity and reproducibility of the resulting small RNA‐Seq and mRNA‐fragment profiles with the type of the RNA (i.e., miRNA, tRNA or mRNA fragments) being a major factor in the choice of isolation method. An interactive web‐based application (miRDaR) with incorporated comparative statistics was also developed to help investigators select the optimal RNA isolation method for their studies (Srinivasan et al., 2019). Results for uEVs demonstrated that when miRNAs are the RNA type under investigation, none of the tested methods has both high reproducibility and high sample complexity, suggesting that choice of (small) RNA extraction method should be driven by the overall small RNA‐Seq data quality metrics to be applied. Interestingly, uEV small RNAs were almost entirely comprised of tRNA fragments (tRFs), and tRF profiles grouped in two clusters based on separation method, suggesting the presence of two major uEV subclasses that carry these small RNAs. For mRNA fragments present in the sequencing libraries, a clear separation of samples based on both sex and type of isolation method was observed, suggesting that gender should be taken into consideration early in study design (Srinivasan et al., 2019).

Capture of uEVs, followed by direct RNA isolation with an optional uEV purification step in‐between and followed by next generation sequencing is common for uEV‐RNA analysis (Mussack et al., 2019; Park et al., 2020). Such approaches can be utilized to minimize sample handling and maximize EV recovery, both of which are attractive for clinical utilization. Acoustic trapping of uEVs followed by RNA isolation and next generation sequencing is another recent example (Ku et al., 2018; Ku et al., 2019). Importantly, a recent study comparing a variety of EV separation methods clearly demonstrates that some widely used methods are not suitable for small and long RNA sequencing, particularly those that combine uEV isolation/separation and RNA isolation (Karina et al., 2020). Thus, it is highly recommended that appropriate pilot studies are performed to assess key performance characteristics of the planned RNA sequencing methods, especially when newly available commercial isolation kits are used. This is particularly vital in studies where small and long RNA sequencing are equally important targets.

A pipeline application for proteomic analysis, including a heat‐shock protein‐based EV capture (Vn96‐peptide ligand) and a subsequent protein fractionation step followed by mass spectrometry was recently described and applied for biomarker discovery in nephronophthisis‐related ciliopathies (Bijnsdorp et al., 2017; Ghosh et al., 2014; Knol et al., 2016; Stokman et al., 2019). A variety of ELISA immunoassay methods exploit unique biophysical features of EVs to facilitate large‐scale and high‐ throughput screening of uEVs for clinical applications (reviewed in (Salih et al., 2016)). Microfluidic devices such as nanoscale lateral displacement arrays on a chip (Nano‐DLD arrays), double filtration microfluidic system on a microchip, microfluidic nanowires followed by in situ RNA extraction, centrifugal lab‐on‐a‐disc nanofilters, and nanoparticle‐based time resolved fluorescence immunoassay (NP‐TRFIA) are prototypes showing the feasibility of isolating and analysing uEVs directly from cell‐free urine (Duijvesz et al., 2015; Islam et al., 2019; Liang et al., 2017; Smith et al., 2018; Woo et al., 2017; Yasui et al., 2017). Important developments allow multiplexing and enable the detection of combinations of markers on the EV surface (Burbidge et al., 2020). In line with this, a single particle interferometric reflectance imaging sensor platform (SP‐IRIS) is now commercially available (Daaboul et al., 2016). A capture chip based on a tetraspanin (CD63, CD9, and CD81) can obtain particle size distribution, images of vesicles and detect up to four different protein markers per EV. These are just few examples applied to the analysis of urine and more information can be found in (Chiriaco et al., 2018; Hartjes et al., 2019; Iliescu et al., 2019). Such technologies are advancing at pace, but none of these have become a consensus standard approach within the community. Uncertainty remains regarding which technology would be the most the optimal system for developing a uEV assay that is truly fit for purpose in clinical diagnostic laboratories.

#### 3.3.2 Direct quantification and characterization of uEVs in cell‐free urine

Reliability of EV separation techniques often correlates with investment of time and money. As stated earlier, all isolation techniques yield a subset of uEVs, which does not necessarily contain all uEVs of interest. Therefore, ideally analysis and assessment of uEVs should be performed on cell‐depleted urine (urine supernatant). Overall, urine analytes are relatively dilute and few platforms are sensitive enough to perform analysis without any pre‐enrichment processing, but quantification and characterization techniques developed for the analysis of cell‐depleted urine are making important progress, and might someday facilitate clinical application of uEVs.

One of the best‐defined techniques for direct quantification and characterization of uEVs is NTA, which can measure particle size distribution and concentration in biofluids (Oosthuyzen et al., 2013). However, NTA (Hole et al., 2013) is prone to user and equipment/software bias, which can further complicate the comparison of multiple datasets. NTA measures all particles present in urine, including protein aggregates, for example, uromodulin or human serum albumin (HSA) aggregates in patients with proteinuria or albuminuria. This can distort the quantification and characterization of uEVs (Gleadle et al., 2018; McNicholas et al., 2017). Conversely, NTA has a lower size detection limit for particles in urine of less than 70 nm in diameter in scatter mode (Oosthuyzen et al., 2013). Thus, NTA may not detect smaller uEVs, which are thought to be the majority (Pisitkun et al., 2004), resulting in an under representation of uEVs. Appropriate resuspension and dilution are necessary since NTA measures clumped particles as a single particle.

Other techniques used to detect and characterize uEVs in cell‐free urine include specialized flow cytometry and TRPS (Coumans et al., 2014). EV flow cytometry uses specific antibodies and/or ligands to either enrich uEVs or exploit the signal of a fluorescent tag linked to the antibody. An example is the use of anti‐tetraspanin coated magnetic beads when analysing EVs with conventional flow cytometry, which offers combined isolation and analysis of uEVs (Campos‐Silva et al., 2019; Welsh et al., 2020). Further developments of flow cytometric based analysis of EVs include use of imaging flow cytometry (Musante et al., 2020) and nano‐flow cytometry (Tian et al., 2020) for direct uEV analysis in cell‐free urine. Whilst relatively new techniques, both of these offer the potential for analysis of individual EVs. Another newly developed assay for the quantification of EVs and detection of multiple biomarkers on the EV surface, without the bias induced by marker dependent EV capture, is EVQuant (Hartjes et al., 2020). In EVQuant, in‐gel immobilization of fluorescently labelled EVs allows high throughput detection of individual EVs and the detection of EV subpopulations and their size distribution (Blijdorp et al., 2021). Another recent technique that could assess the global composition of uEVs at the single particle level or in a limited group of EVs is Raman Tweezers microspectroscopy (RTM) which could help to determine the percentage of different EV subpopulations and contaminants present in the preparation (Kruglik et al., 2019).

In general, all EV analysis approaches and assays currently developed are hampered by the small size and large heterogeneity of EVs in bio‐fluids. Improvements in sensitivity and specificity are needed to truly access the whole range of EVs and EV subpopulations in both research and clinical applications.

### 3.4 Normalization

In order to effectively maintain water and salt homeostasis, urine production can be highly variable. Consequently, the concentration of EVs in urine may vary more than in blood and other body fluids. In addition, uEV processing protocols invariably induce additional variation that may need to be corrected for (Momen‐Heravi et al., 2012; Yuana et al., 2011). Thus, a major challenge of uEV research is the lack of robust methods to normalize uEV content to adjust for confounding factors such as excretion rate and uEV‐processing‐related variation (Blijdorp & Hoorn, 2019). Normalization approaches for urine biomarkers can be broadly classified as calculating an absolute or relative excretion rate. **Relative excretion rate** defines the abundance of the uEV marker in relation to another marker, such as uEV number, a protein or RNA marker, or total EV protein, RNA or lipid amount (Table 4). This is most commonly applied in urologic and in proteomics studies but also used in kidney‐related studies (Bijnsdorp et al., 2017; Chen et al., 2012; Dhondt et al., 2020; Dijkstra et al., 2014; Gonzales et al., 2009; Oosthuyzen et al., 2013; Pisitkun et al., 2004; Samsonov et al., 2016; Silvers et al., 2016). **Absolute excretion rate** defines the rate (per unit of time) in which a uEV marker is excreted. This can be measured using a timed collection, or may be approximated by normalization to urine osmolality or creatinine in a spot urine (Adedeji et al., 2019; Nisell et al., 2006). This is mostly used in kidney‐related research, and is of particular importance in physiological studies (Salih et al., 2016; Blijdorp et al., 2021).

#### 3.4.1 Relative excretion rate

A commonly used normalization strategy for proteomic analyses is to start with a reproducible method to enrich for uEVs and conduct the experiment with the same **amount of total protein** (e.g., 20 μg) per sample. After acquisition, protein data can be processed using quantile normalization, which assumes that the majority of proteins present in the sample are stable. Protein variation in uEVs has been recently determined by Oeyen, *et al*. (Oeyen et al., 2018). Such global normalization approaches (e.g., Linear scaling to Counts Per Million) are also applicable to transcriptomics studies and were recently demonstrated for small RNA‐Seq data generated from EVs in different biofluids, including uEVs (Srinivasan et al., 2019). Nevertheless, the effect of different normalization approaches, in particular for long transcripts, remains to be systematically evaluated. Importantly, in urine, uromodulin and albumin are known to be overrepresented in protein content after uEV enrichment protocols (Fernandez‐Llama et al., 2010; Musante et al., 2012; Xu et al., 2019).

Expression of uEV‐biomarkers as a ratio to **uEV number** or to a **uEV‐biomarker** (e.g., a housekeeping control transcript or a protein, present in uEVs) that is considered to be stable in the studied condition has been also proposed (Colombo et al., 2013; Rodriguez et al., 2017). However, such ratios can be affected by the quality of the chosen control(s) in terms of expression stability. In addition, common EV‐markers such as CD9 or CD63 may be differentially expressed throughout the urogenital system, and therefore not be generally applicable on urine samples (Blijdorp et al., 2021). External factors such as an undetected infection or damage/injury/inflammation in any part of the urogenital system affect the total excretion of uEVs and the composition of the uEV pool (Duijvesz et al., 2015; Hendriks et al., 2016).

#### 3.4.2 Absolute excretion rate

**Timed collection**, and in particular 24‐hour collection (i.e., during an exact 24‐hour time‐course discarding first morning void and including first morning void of following day) is considered the gold standard to determine excretion rate of general urinary biomarkers such as albumin, because it is less sensitive to fluctuations due to circadian rhythm (Koopman et al., 1989). However, 24‐hour urine collections are time consuming and impractical for the patient and can lead to collection errors (Boyd et al., 2018). Moreover, prolonged collection of uEVs may accelerate their degradation (Oosthuyzen et al., 2013), although this remains under debate (Mitchell et al., 2009). The measurement of an absolute excretion rate of a urine biomarker using a timed collection can be approximated in a spot urine measurement by a **ratio to urinary creatinine** (Gunasekaran et al., 2019), which has been shown to be highly effective for both intra‐individual comparison (96% of uEV variation explained by creatinine concentration) and inter‐individual comparison (47–82%) (Blijdorp et al., 2021). Creatinine is a waste product of muscle catabolism. In the healthy kidney, the excretion rate of creatinine is constant when the glomerular filtration rate, secretion by organic cation transporters, and body muscle mass do not change. Thus, the ratio to creatinine should be validated in acute kidney injury or different stages of chronic kidney disease (Forni Ogna et al., 2015; Waikar et al., 2010). In addition, comparing individuals may need correction for creatinine excretion or muscle mass. **Urine osmolality** has also been applied as an alternative urine normalization factor in targeted metabolomics (Khamis et al., 2018). Urine osmolality assumes there is constant excretion of osmoles in steady state, which was shown not to be the case during water loading (Blijdorp et al., 2021). Ginsberg, *et al*. (Ginsberg et al., 1983) show that the protein/creatinine ratio of single void urine collected after the first voided morning specimen and before bedtime best correlates with the quantity of protein excreted during 24 h.

#### 3.4.3 Normalization to organ‐specific biomarkers

In some cases, organ‐related biomarkers can be utilized for normalization. For example, in studies addressing prostate‐derived EVs, the **urinary Prostate Specific Antigen** (uPSA) can be used as a measure of the amount of prostatic fluid released in the urine and as a surrogate marker and normalization factor for the number of prostate‐derived uEVs (Duijvesz et al., 2015; Minciacchi et al., 2017; Ploussard & de la Taille, 2010). CD24, a kidney‐specific uEV marker, could possibly be used as a reference for kidney‐derived EVs (Keller et al., 2007). While normalization to GFR or nephron mass has not been used in the literature, it may improve results of studies concerning the kidney.

## 4 RECOMMENDATIONS AND CONSIDERATIONS

### 4.1 Urine collection and biobanking for uEV research

Biobanking of urine is crucial for future biomarker studies. Academic institutions, hospitals and professional biobanks worldwide often share biobanking protocols. However, collection, processing and storage methods as well as the extent of gathered sample/donor information differ greatly between sites. As specific biobank guidelines covering all uEV research have not been established and EV‐dedicated biobanks/collections are rare, it is recommended to follow the general recommendations related to the collection, storage, preprocessing and transportation of the urine samples by the authorities in the urine analysis field, including the Clinical Laboratories and Standard Institute (CLSI) (Rabinovitch et al., 2009). It is important to be aware of the preanalytical variables and follow as much as possible, the recommendations for their reporting summarized in (Table 2).

Based on the expertise existing among the actual members of the ISEV Urine Task Force some recommendations for uEV research can be given. However, it should be clarified that this is a rapidly evolving field and that the recommendations are part of an ongoing work. Moreover, at this stage these recommendations do not represent the view of all the uEV researchers.

- When starting research with **existing biobank** samples collections, gather all available sample and donor related data for reporting and analysis purposes.
  - Dipstick data can be gathered after thawing to indicate the presence of interfering or abnormal components.
- When **starting a new urine collection or a biobank**, consider and record the parameters in the whole logistics chain from donor recruitment to data management and urine collection, transport, preprocessing, aliquoting and storage. It is safest to consider the broadest possible future uses of the urine and uEV samples.
  - Keep collection, processing, and storage procedures the same throughout the study. If this is not possible, perform controls to identify the possible effect of the varied step.
  - Aim for fast processing (hours), keep samples cold (+4°C, ice or equivalent) and consider additives (e.g., azide, protease inhibitors, EDTA) to avoid microbial growth and maximize preservation of the EVs.
  - Gather dipstick data to indicate the presence of interfering or abnormal components.
  - Centrifuge urine before freezing to remove cells that could be disrupted during freezing. Aliquot samples according to future use and available space. Freeze at ‐70°C or colder.
  - Use only hygienic collection devices, containers and plastics that resist urine pH and do not bind uEVs (lipids/proteins) or shed particles.

It is also important to collect and report low evidence level items to improve our understanding of the impact of these factors and reduce current uncertainties. These may include:

-  Need for light protection or for some sample protecting agents, such as RNase inhibitors or cryoprotectants.
-  Freezing speed. Quick freezing appears to work, but tests for a range of freezing speeds are lacking.
-  Defrosting temperature.

### 4.2 Downstream analysis of uEVs

**FIGURE 4:**
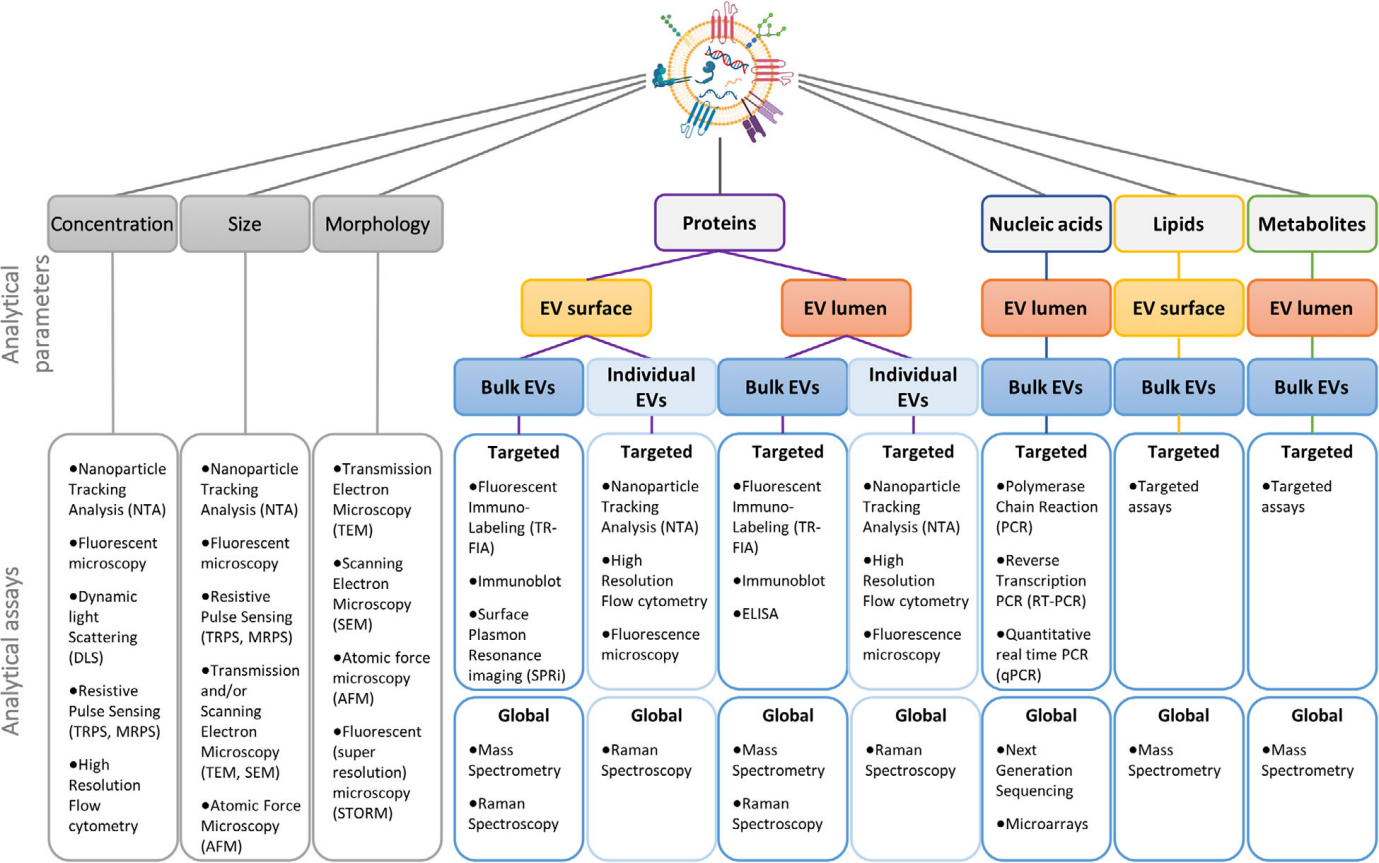
Analytical method selection in uEV research. Analytical methods used for the characterization of EVs explore their physical properties (grey) and/or molecular components (colour). Commonly studied molecular components found in EVs are proteins, nucleic acids, lipids and metabolites. Localization of these molecular components largely defines the choice of an analytical approach. Proteins (purple) can be localized in the EV membrane or lumen. EV surface proteins can be assessed specifically by antibodies, both in bulk analysis, for example, by a time‐resolved fluoroimmunoassay (TR‐FIA), Immunoblot, immuno‐bead capture‐based flow cytometry, or surface plasmon resonance imaging (SPRi) and with assays that analyse individual EVs such as fluorescent NTA, high‐resolution flow cytometry and microscopy. Analysis of luminal proteins can be performed in bulk assays, for example, immunoblot, ELISA and time‐resolved TR‐FIA after membrane permeabilization. Generally, labelling of luminal cargo can facilitate individual EV analysis through the use of membrane‐permeable fluorescent dyes that label proteins or nucleic acids such as ExoGlow™ or Syto™13. Whilst such dyes lack the specificity of more targeted approaches, they enable analysis of EVs by fluorescent microscopy, fluorescent NTA, and high‐resolution flow cytometry. Specific analyses of nucleic acids (blue) and metabolites (green), generally considered to be luminal, are usually achieved in bulk EV assays by either omics‐based approaches, or by transcript‐specific PCR based techniques. Lipids (yellow), are localized within the EV membrane and are commonly analysed in bulk assays either by mass spectrometry or colorimetric reagents, like the sulfo‐phospho‐vanillin (SPV) lipid assay

As with most body fluids, urine contains EVs from a plethora of different organs, tissues and cell types from the urinary tract (Figure 2). Together with the wide variety of analytical parameters that can be obtained from EVs, this results in several important considerations for the analysis of uEVs (Figure 4).

The first consideration is the **type of analytical parameter** that is going to be studied. uEV analysis can be focused on physical parameters (e.g., concentration, size distribution, morphology) and/or the biochemical content of uEVs (e.g., proteins, nucleic acids, lipids and metabolites). This is reflected by the wealth of state of the art and newly emerging EV assays and analysis technologies available (Hartjes et al., 2019; Nazarenko, 2020; Paisrisarn et al., 2020; Skotland et al., 2020; Soekmadji et al., 2020; Williams et al., 2019).

However, there is no single consensus protocol for pre‐processing EVs, or analytical technology that suites most or all analytical parameters. Importantly, the MISEV2018 and EV‐TRACK guidelines recommend to report on several complementary analytical parameters (e.g., concentration, size distribution, morphology, EV markers) to confirm the presence of EVs (EV‐TRACK Consortium et al., 2017; Thery et al., 2018).

The requirement of pre‐analysis **separation and purification** of uEVs is essential for many of the (biochemical) analyses to avoid interference of non‐EV contaminants in urine, but might be nonessential or maybe even disadvantageous for other analyses as any isolation or purification protocol unavoidably leads to significant loss of EVs and EV material. In addition, isolation procedures are generally biased towards certain EV size and density ranges. It is therefore recommended to avoid EV isolation or purification protocols as much as possible (except for the pre‐freezing clearing as described in section 3.1.2.6) and only implement extensive EV isolation and purification when needed due to interference by other components of urine (Vergauwen et al., 2017; Wachalska et al., 2016; Xu et al., 2019). Direct analysis of uEVs without time‐consuming and costly extensive pre‐processing would be highly beneficial for clinical implementation. However, when EV isolation is required, different approaches (e.g., ultracentrifugation and precipitation) should be evaluated for urine as specific biofluid and the analytical parameter of choice (Coughlan et al., 2020; Mussack et al., 2019; Oeyen et al., 2019; Paisrisarn et al., 2020; Royo et al., 2016; Wang & Sun, 2014). Regarding estimates of size and concentration, different techniques can be applied. While NTA and TRPS offer particle counting and sizing including non‐EV particles, flow cytometry for example, can offer single EV detection and might be more precise. To our knowledge studies are needed to understand if counting with these techniques are suitable for normalization.

A third consideration is a result of the wide variety of organs, tissues and cell types that contribute to the uEV pool. Depending on the scientific or medical question being asked, **initial (on‐assay) capture of specific uEVs of interest** (e.g., uEVs derived from specific organs or diseased tissue) can enhance the specificity and sensitivity of the analysis. Such capture within the analytical assay relies on the availability of suitable capture targets on the EV surface and the efficiency of capture. Moreover, the yield of specific uEVs in these capture approaches could be a concern. Capture based assays often use the (so called) general and abundant EV surface markers CD9, CD63, and CD81 for capture, for example, time‐resolved fluoroimmunoassay (TR‐FIA), surface plasmon resonance imaging (SPRi), ExoView® (Daaboul et al., 2016; Duijvesz et al., 2015; Rikkert et al., 2020). However, it has become increasingly apparent that only fractions of EVs carry these ‘general’ EV markers, and that expression of these markers is largely dependent on the cells of origin (Kowal et al., 2016; Salih et al., 2016).

The need for capture of tissue or disease specific EVs can be overcome by analysis of **individual uEVs rather than bulk analysis**. The analysis of individual EVs allows the identification and subsequent characterization of specific uEV subtypes without the need for specific isolation. For example, multiplexing strategies allow the analysis of multiple EV surface markers on individual EVs (Hartjes et al., 2020; Headland et al., 2014), sometimes after capture of the EVs (Daaboul et al., 2016; Koliha et al., 2016; Wiklander et al., 2018). This is again dependent on the availability of specific EV (surface) markers that can be used for detection. Moreover, the analysis of individual EVs is currently restricted to measuring physical parameters like concentration, size and morphology, as well as proteins on the EV surface and lumen. Super‐resolution imaging for instance may enable visualization of structure, biomarker distribution, and relative abundance of each biomarker on single EVs. Technologies to analyse RNAs, DNA, lipids, and metabolites in individual EVs are not yet available.

The level of EV analysis varies from global, discovery based approaches using the ‘omics’ family of technologies (e.g., proteomics, transcriptomics, genomics, lipidomics, and metabolomics) (Cheng et al., 2014; Lee et al., 2018; Park et al., 2020; Rigau et al., 2013; Wang et al., 2020), to more targeted analysis of specific EV contents using immune detection or PCR‐like approaches to measure specific proteins or RNAs of interest (Samsonov et al., 2016; Sole et al., 2019; Sun et al., 2012; Yamamoto et al., 2018). The latter is more present in target‐specific EV assays and is more suitable for clinical implementation. Analytical technologies and assays for these two levels of uEV analysis differ and require different levels of pre‐processing and purification.

The last consideration is the requirement of **scalability**. Many current technologies for the analysis of (individual) EVs require individual samples be measured independently. Large‐scale experiments and studies on larger cohorts of uEV samples will require more high/medium throughput technologies. To support the scalability of uEV analysis, several technologies are being developed that enable higher throughput using automation and miniaturization of assays in (microfluidic) devices. Related to scalability is standardization. At this moment, many of the analytical assays for EVs are highly dependent on details in the protocols and settings. It is therefore pivotal to introduce optimal levels of standardization and reporting in the analysis of uEVs to improve reproducibility (EV‐TRACK Consortium et al., 2017; Thery et al., 2018).

#### 4.2.1 Analysis of the uEV proteome

Many of the potential challenges of working with uEVs highlighted elsewhere in this manuscript also apply to proteomic analysis of uEVs, especially those relating to vesicle isolation and purity (section 3.2). Abundant proteins in urine such as uromodulin, previously reported to be present in uEVs (Pisitkun et al., 2004), may in fact be co‐isolated or partially related to EVs that have been co‐isolated with uEVs (Musante et al., 2020). Moreover, problems associated with a high abundance of soluble proteins are exacerbated in various clinical scenarios such as proteinuria, hematuria, and other conditions. Therefore, one must be careful when analysing complex data sets from broad proteomic studies of uEVs. Whilst additional techniques can be used to remove soluble proteins from the sample, it remains a challenge to distinguish proteins that are genuinely uEV‐associated from soluble contaminants. Furthermore, issues with protein contaminants make normalization based on vesicular proteins extremely difficult. An alternative approach is to normalize sample inputs based on vesicle count. The challenges associated with either approach are summarized in section 3.4. There have been several advances in technologies for focused analysis of the uEV proteome. Technologies such as aptamers or proximity extension assays (PEA) have been utilized for analysis of EV proteins (Larssen et al., 2017; Welton et al., 2016; Zhu et al., 2020). Such techniques offer greater sensitivity and limit the background noise which may accompany traditional mass spectrometric approaches, but the breadth of analytes assessed is limited. Additional approaches utilizing immuno‐based capture and detection of proteins can also be used for assessment of selected uEV proteins. Low density array (LDA) profiling can be adapted for the study of vesicular proteins (Cha et al., 2018; Mata Forsberg et al., 2019). Whilst such arrays are limited in their coverage, they do not require access to specialized equipment. In addition, there are several commercially available platforms for assessment of multiple uEV surface markers in plate‐ or chip‐based formats (Gori et al., 2020; Musante et al., 2017). However, such immuno‐affinity assays are susceptible to soluble protein contaminants that can interfere with uEV capture and detection. A comparison of techniques for uEV protein analysis described above is shown in Table 4.

#### 4.2.2 Analysis of the uEV transcriptome

RNAs carried by uEVs are biologically active, can reflect the physiological status of cells of origin, and have been intensely studied in the search for biomarkers (Peinado et al., 2012; Valadi et al., 2007). Characterization of the RNA species in uEVs depends on the preanalytical and analytical conditions. The RNA yield from uEVs is related to the uEV separation technique used (e.g., 2.6–50 pg/ml for uEVs isolated by ultracentrifugation followed by 0.1 μm filtration) (Bryzgunova et al., 2016), and 17–46 pg total RNA per million uEVs obtained by UC alone (Royo et al., 2016). An extensive description of analytical conditions for RNA analysis was recently reviewed (Everaert et al., 2019). Furthermore, microfluidic techniques have been developed to reduce bias introduced by high manipulation of the sample for targeted detection (Yasui et al., 2017). A comparison of techniques for uEV RNA analysis is shown in Table 5.

#### 4.2.3 Analysis of the uEV lipidome

Preanalytical and analytical parameters can affect outcomes of EV lipid analyses and should be reported (Avela & Siren, 2020; Gori et al., 2020; Wu et al., 2020). Protocols for sample preparation, lipid extraction, and separation must be reproducible. For example, it is not clear yet to which extent uEV lipids can be degraded under different conditions. Moreover, the presence of lipoparticles in EV samples can affect lipid analysis, which should be considered in studies of conditions that can lead to an increased lipid concentration in urine. Recent studies of the EV lipidome have often used mass spectrometry. Because of the high molecular diversity of lipids, overlaps of mass spectrometric ions of lipid species frequently occur. Therefore, using high‐resolution MS is recommended for analysis of the uEV lipidome (Zullig & Kofeler, 2021). In addition, proper internal standards, normalization and/or labelling are crucially required for precise quantitative lipidomics of uEVs (Avela & Siren, 2020; Glover et al., 2019; Tipthara & Thongboonkerd, 2016; Wu et al., 2020; Zullig & Kofeler, 2021).

#### 4.2.4 Analysis of the uEVs metabolome

uEVs carry different types of metabolites such as many organic acids involved in the tricarboxylic acid cycle, bile acids, amino acids, nucleotides, and steroid hormones pointing to these vesicles as indicators of the metabolic status of tumour tissue (Clos‐Garcia et al., 2018; Puhka et al., 2017; Royo et al., 2016). However, several issues exist with the analysis of EVs by MS‐based metabolomics. The technique is very sensitive, and it is likely that some non‐EV metabolites will be retained by most EV separation methods. Background metabolites can be assessed easily for cell culture conditions by analysing unconditioned medium (Palomo et al., 2014; Royo et al., 2019), but it is not as easy to judge background metabolites for urine. Therefore, it is recommended to study multiple biological replicates and take into consideration only those metabolites that are consistently detected among technical replicates and samples (Clos‐Garcia et al., 2018). Another aspect to consider is that a minimum amount of uEVs will be required to obtain reliable measurements, for example, 50 micrograms of total uEV protein. Finally, the varied chemical nature of the metabolites in uEVs means that there is no single method capable to analyse all uEV metabolites at once. A combination of different extraction methods chromatographic parameters and mass spectrometric conditions are likely needed to construct a complete picture of the uEV metabolome.

### 4.3 Normalization of uEV data

Normalization approaches for urine biomarkers can be broadly categorized as absolute or relative excretion rates. The r**elative excretion rate**, is generally applicable as a normalization method for uEV samples subjected to any isolation protocol while the **absolute excretion rate** is ideally used with techniques that characterize uEVs directly in cell‐depleted urine. Without a universal approach to normalize uEV samples, we list here current normalization methods in use:

-  Timed collection (gold standard: 24‐hour collection) – absolute excretion rate
-  Creatinine/osmolality normalization – estimate of absolute excretion rate using spot urine
-  Constitutively expressed uEV marker – relative excretion rate
-  Specific marker ratio (e.g., organ specific proteins) – relative excretion rate
-  Relation to total uEV count – relative excretion rate
-  Z‐normalization (RNAseq/MassSpec) ‐ relative excretion rate
-  To GFR (or nephron number) – relative excretion rate (organ‐related: kidney)
-  Relation to PSA (e.g., after DRE) – relative excretion rate (organ‐related: prostate)

The strengths and limitations of each normalization method are mentioned in Table 3.

**TABLE 3:** Normalization methods

Normalization method	Application	Strengths	Limitations
Constitutively expressed uEV biomarker	Relative excretion rate	Adjusts for isolation variability or incomplete uromodulin depletion Simple normalization rationale Possible surrogate measure for EV number (requires further validation)	Currently limited to proteins Biomarker not always valid for the analyte of interest Affected by changes in (external) excretion of biomarker from any part of the system (e.g., urothelial release when studying kidney disease) Some EV biomarkers may not be as universal as originally believed
Relation to total uEV quantity	Relative excretion rate	Adjusts for isolation variability or incomplete uromodulin depletion Simple normalization rationale Adjusts for changes in general EV release	Problematic if change in total excretion of uEVs is part of underlying pathology (e.g., after nephrectomy) Highly dependent on the method of uEV characterization Affected by change in (external/crossover) EV secretion from any part of the system (e.g., urothelial release when studying kidney disease)
Specific biomarker ratio: ratio of two or more (disease) related biomarkers, ideally with a (known) similar source	Relative excretion rate	Adjusts for isolation variability or incomplete uromodulin depletion Can leverage mechanism of action of biomarkers, especially when they go in opposite directions Less sensitive to external/crossover secretion of uEVs	Depends on the existence of a biomarker ratio that steadily predicts an outcome Often high variability Each ratio should be independently validated
Mass spectrometry proteomics; Z‐ or quantile normalization	Relative excretion rate	Adjusts for isolation variability Uses all protein information available to normalize content – less sensitive to external/crossover factors provided they are small	Albumin and/or THP can dominate the uEV proteome and can vary more than other uEV proteins Affected by change in (external) EV secretion from any part of the system (e.g., urothelial release when studying kidney disease)
RNAseq; Z‐ or quantile normalization	Relative excretion rate	Adjusts for isolation variability Uses all RNA information available to normalize content – less sensitive to external/crossover factors provided they are small	May be biased when comparing two different patient groups Affected by change in (external) EV secretion from any part of the system (e.g., urothelial release when studying kidney disease)
Timed collection (ideally 24 h)	Absolute excretion rate	Compare intra‐ and inter‐individual differences without further normalizations Eliminates variability due to circadian rhythm	Inconvenient Often incomplete collections Long processing time increases chances of sample degradation Does not adjust for possible variability in uEV processing protocols Consider longer cycical variation periods (e.g., changes over several days or even weeks)
Urine creatinine/osmolality	Measure of absolute excretion rate in random spot urine	Commonly used clinically Easy and inexpensive to assay May correct for circadian rhythm in GFR	Differences or changes in muscle mass/creatinine excretion require correction Does not adjust for possible variability in uEV processing protocols, or circadian rhythm in uEV release. Requires further validation in uEVs
GFR/nephron number	Excretion relative to kidney size	Commonly used clinically (GFR) May help to compare patients with different stages of kidney disease	Non‐invasive methods to estimate nephron number are unreliable Requires validation in uEVs
Urinary PSA	Excretion relative to prostate size	Commonly used clinically (PSA) Easy to assay	Requires further validation in uEVs

**TABLE 4:** Summary of techniques for EV proteome analysis

Technique	Required sample input	Strengths	Limitations	References
Mass spectrometry	High	Broad spectrum of analytes Non‐biased Well established protocols	Susceptible to “noise” from contaminants Data requires trimming/cleaning	(Conde‐Vancells et al., 2010; Pisitkun et al., 2004; Welton et al., 2010)
Aptamers	Medium	High sensitivity High specificity Can measure 1000 s of analytes Focused	Limited coverage (analytes assessed: 1000 s)	(Welton et al., 2016; Zhu et al., 2020)
Proximity extension assays (PEA)	Medium ‐ low	High sensitivity High specificity Focused High throughput	Severely limited coverage (analytes assessed: 100 s)	(Larssen et al., 2017)
Proteome Profiler Arrays	Medium	Focused No specialist equipment required Relatively low cost	Minimal coverage (analytes assessed: 10 s) Low dynamic range Potential interference to immuo‐capture by soluble contaminants	(Cha et al., 2018; Mata Forsberg et al., 2019)
Immuno‐affinity assays (high‐resolution flow cytometry, chip/plate‐based analyses)	Minimal	Focused Relatively low cost Versatility	Minimal coverage (analytes assessed: 10 s) Potential interference to immuno‐capture by soluble contaminants	(Gori et al., 2020; Musante et al., 2017; Rikkert et al., 2020)

**TABLE 5:** Summary of techniques for EV RNA analysis

Technique	Strengths	Limitations	Comments	General Recommendations	Particular Recommendations
**RNA‐seq** Describes quantity and sequences of RNA using NGS	‐ Detection of low and high expressed genes ‐ Detection of isoforms/splice variants ‐ Detection of new sequences ‐ High sensitivity ‐ Identifies different RNA species in one analysis (coding and non‐ coding) ‐ Raw data can be used by different researchers to make new analysis.	‐ Cost ‐ Training for data analysis ‐ Data management and storage ‐ Small amount of reference databases. ‐ Lack of internal controls ‐ The RNAs described by the analysis depends on the database used.	‐ RNA can be isolated as total RNA or small RNAs by using different RNA isolation kits, before library construction. ‐ Different libraries can be created previous to NGS to enrich and/or deplete RNA populations (important in samples with low starting material): Whole transcriptome, targeted transcriptome (10 ng), targeted RNAs (500 pg–5 ng), small RNAs. ‐ Data analysis parameters, raw data, pre‐ and analytical conditions should be available to compare between different studies	Preanalytical: ‐ Centrifugation of urine upon receive to remove cells, manage at 4°C to avoid cell rupture and microbial contamination. ‐ Cell free urine as starting material. ‐ Long term storage of cell free urine at ‐70°C ‐ Reporting pre‐ analytical conditions according to MISEV2018 guidelines. Analytical: ‐ Organic extraction increases RNA yield ‐ RNA extraction method must be reported ‐ Share raw data in public databases (EV‐ TRACK, Exocarta, etc.)	Preanalytical: ‐ uEV isolation method: All methods available to date works well Analytical: ‐ Library construction must be reported Data analysis: ‐ Describe data analysis parameters
**RNA array** Describes quantity of predefined RNA sequences	‐ Easier data analysis ‐ Less data storage required ‐ Detects expression of a set of predefined transcripts ‐ High amount of reference databases	‐ Detection of highly expressed genes. ‐ Depends on the affinity of the probes.	‐ RNA can be isolated as total RNA or small RNAs by using different RNA isolation kits.		‐ Use multiple probe sets per target
**qPCR** Describes quantity of predefined RNA sequences	‐ Low cost for processing and implementation ‐ Low starting material	‐ Lack of normalization parameters ‐ Depends on the affinity of the probes.	‐ Targets can be obtained from RNA‐seq data		Preanalytical: ‐ When based in RNA‐seq data, process sample under the same conditions Analytical: ‐ Add synthetic RNA sequences to starting material to normalize ‐ Use same volume of starting material ‐ Characterize the reproducibility of the expression of internal controls

Alvarez et al., 2012; Bryzgunova et al., 2016; Everaert et al., 2019; Khurana et al., 2017; Langevin et al., 2020; Mussack et al., 2019; Park et al., 2020; Rao et al., 2018; Royo et al., 2016.

Important criteria for developing new normalization tools are:

-  Decreases variation within normal or expected range
-  Widespread availability and feasibility
-  Can be validated internally and across testing sites, ideally with (shared) external standards
-  Compatibility with commonly used isolation and/or analysis methods.

### 4.4 Functional studies of uEVs

#### 4.4.1 General recommendations for uEV functional studies

Common issues and general recommendations to be considered for attributing a functional activity to EVs are extensively detailed in MISEV 2018 (Thery et al., 2018). Therefore, refer to the MISEV 2018 guidelines for the design of experiments evaluating functional activities of uEVs or uEV subtypes. Here, we briefly summarize the most relevant points of interest:

-  Possible artifacts due to EV contaminants should be excluded. This can be achieved by comparative evaluation of the effect of the biofluid of interest before and after EV removal, together with that of the isolated EVs; when possible, the main contaminants must be isolated and their effect tested as well. Moreover, the role of co‐isolated non‐EV material should be studied using (combined) enzymatic degradation of proteins or RNA/DNA species to allow investigations addressing the “EV‐corona” (Palviainen et al., 2020). In particular, low dose trypsin, proteinases, RNAses and DNAses might be useful. Appropriate protocols should be optimized in order to avoid EV disruption or degradation in the same time.
-  Isolation of the crude EV population and, when of interest, of the different EV fractions, should be achieved using multiple and accurate methods. To ascribe a functional property to specific fractions, side‐by‐side analysis of all fractions is recommended.
-  Appropriate controls should be included such as unrelated EV sources and disease EV controls such as healthy, untreated or otherwise matched donors,
-  Functional activity should be quantitatively related to the amount of EVs or of a specific EV component; this can be achieved by EV normalization strategy supporting comparison of different EVs, fractions and active cargo and possibly by the evaluation of dose response effects.

#### 4.4.2 Specific considerations for uEV functional studies

While the fundamental practical considerations detailed above must be applied to all functional analysis studies (regardless of the source of EVs), urine presents certain specific challenges that must be considered when evaluating the functional activity of urine EVs. As detailed earlier, the timing and type of collection method may lead to dramatically different levels of cellular elements, including EVs in urine samples. Thus, when possible, the same collection method should be used for any comparative analysis. In addition, uromodulin can entrap small EVs in polymer “nets” and reduce recovery. Releasing EVs from uromodulin is therefore necessary to avoid a biased functional analysis which focuses on a small subset of urinary EVs. However, complicating this is the fact that procedures which disrupt the uromodulin network and release EVs (i.e., DTT (Fernandez‐Llama et al., 2010) may also lead to co‐elution of uromodulin in the EV pellet. As uromodulin is well known to modulate a diverse array of processes (i.e., immune function, sodium handling, complement system (Olinger et al., 2019)) one must consider whether co‐eluted uromodulin is responsible for any effects attributed to EVs. Similarly, as bacteria may also co‐elute in EV isolation procedures, one must consider the possibility of bacterial contamination in urine samples. This could also lead to biological activity that is incorrectly attributed to EVs. One strategy to address this may be to assess contamination after collection and discard contaminated samples (Hogan et al., 2015), however this is not practical for all applications. Ultimately the task force recognizes that functional analysis of uEVs is very early in its evolution and identification of strategies to address the above challenges should be a research priority.

## 5 FUTURE PERSPECTIVES

### 5.1 Clinical challenges

**FIGURE 5:**
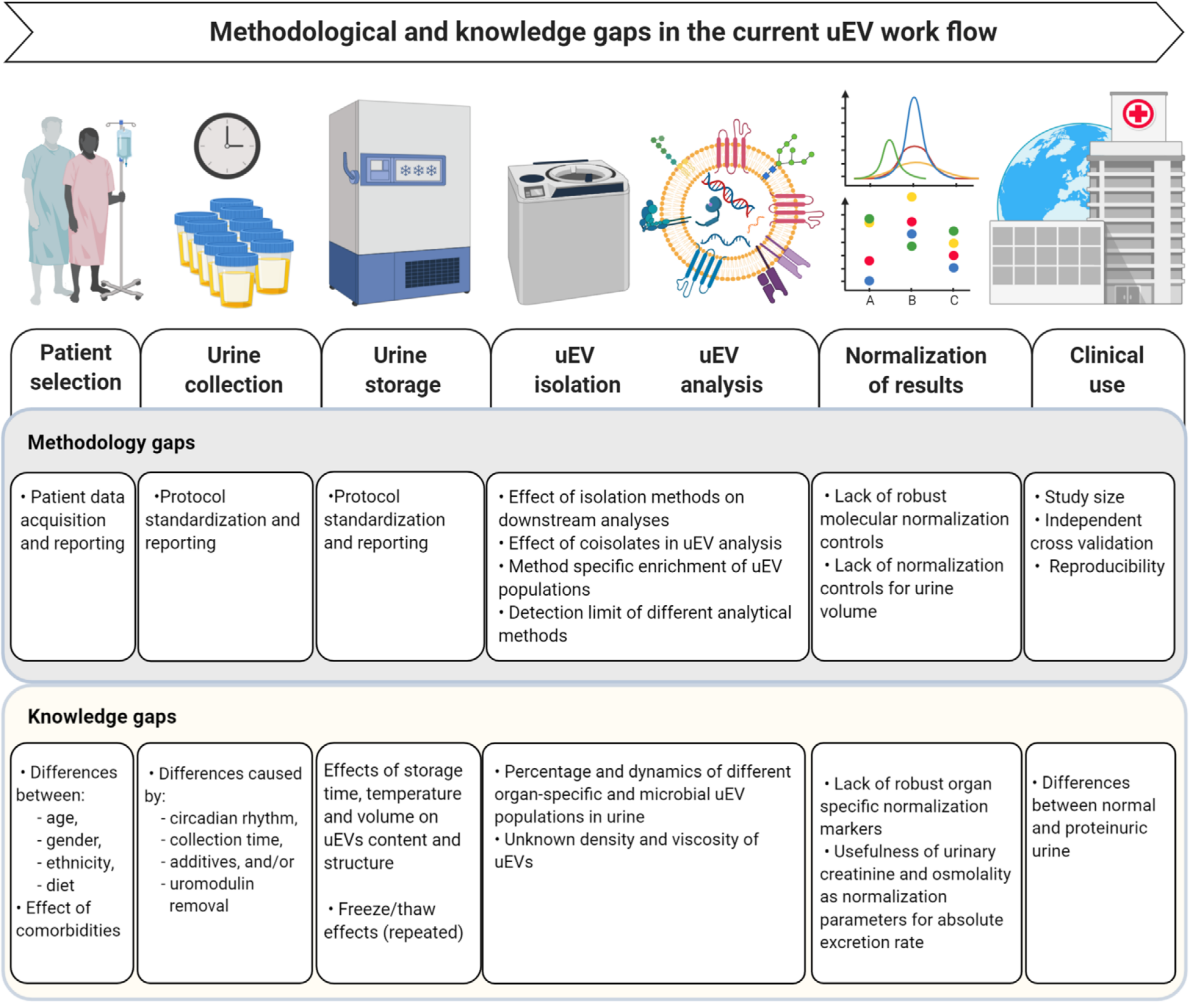
Methodological and knowledge gaps in the current uEV work flow. The urine EV task force of the International Society for Extracellular Vesicles is in the process of recruiting uEV researchers to perform collaborative studies of rigor and reproducibility to address the outlined knowledge gaps

Use of uEVs as novel biomarkers for diagnosis, prognosis, and guidance for treatment also has its challenges. The uEVs research community faces several gaps that should be overcome to systematically advance the field (Figure 5). Validation studies are needed to show superiority of uEV‐shuttled biomarkers to direct measurement of the protein/RNA/lipid biomarker of interest in urine, that is, is there a genuine advantage to concentrating uEVs. It is also important to note that a single standardized approach for urine collection, uEV separation and measurement has not yet been adopted and likely will not be. The impact of different pre‐analytical variables on the nature and quality of uEV isolates has to be understood in order to design, optimize and escalate protocols towards real‐world clinical applications. Use of uEVs from existing biobanks also represents a clinical challenge because the standardization necessary for many assays may be insufficient or different compared with what is needed for uEV assays. An additional challenge in the field relates to normalizing biomarker signals (Gunasekaran et al., 2019) because urine is one of the most dynamic biofluids. In order to move the field of uEV research forward, uEV reference standards are needed for many experimental purposes, including single EV analysis, for example, for flow cytometry and particle analysers for assessment of size and concentration or normalization to excretion rate and uEV processing‐related variation.

Among the many issues mentioned herein is the overriding need for more cost effective and tractable assay approaches that can provide fast quantitative information in a standardized fashion. Currently, the technologies available for EV analysis are highly diverse and somewhat idiosyncratic. Many of these platforms have limited accessibility, residing within specialized laboratories or within companies providing analytical services based on their proprietary technologies. Although healthcare systems globally operate differently, development of uEV biomarker measurement technologies that can be broadly deployed to diagnostic centers, for example, within hospitals, will be needed to fully realize the biomarker potential of uEVs (Rayyan et al., 2018). These are not trivial issues and will require continuous collaborative discussions involving industry, regulatory bodies and standards agencies to ensure success.

### 5.2 Clinical potential of uEVs

Currently the diagnosis of many diseases of the kidney and urinary tract are based on insensitive and non‐specific biomarkers. For instance, changes in kidney function are still measured using changes in serum creatinine – a late and nonspecific marker of kidney dysfunction (Thomas et al., 2015). Despite years of intense research, there are only a few biomarkers approved for clinical use. Examples include tissue inhibitor of metalloproteinase 2 (TIMP2) and insulin‐like growth factor‐binding protein (IGFBP7), urinary biomarkers for acute kidney injury (AKI) incorporated in a commercial test (Nephrocheck) (Fan et al., 2019; Kashani et al., 2013). Even this FDA‐approved test is falsely positive in 50% of people without AKI (Gaffney, 2014) pointing to a clear requirement for a new approach to identify and measure fit‐ for purpose disease markers. Early identification of disease processes in the kidney and urinary tract is clearly needed to improve the specificity of diagnosis, facilitate earlier and better tailored interventions and ultimately for improved outcome for patients.

Urinary EVs hold excellent potential as a multiplex‐biomarker source. They are easily accessible **non‐invasively, available in large quantities, and amenable to frequent longitudinal sampling**. uEVs in part resemble the molecular content of the parent cells from which they are released (Bazzell et al., 2018). They carry cell specific markers from every segment of the nephron and urogenital tract and therefore are ideal for sampling the health status of these systems. Moreover, reports of EVs arriving into the urinary system from distant sites such as in lung cancer (Fraser et al., 2016; Li et al., 2011) are important, as they highlight the potential for identifying diseases in unrelated organ systems through urinary sampling. These are avenues ripe for future exploration and development, potentially establishing uEVs as the ultimate biomarker source.

It is also increasingly recognized that improvements in the diagnosis, prognosis and treatment of disease processes require a better understanding of distinct underlying cellular and molecular mechanisms. Therefore, researchers in this field are exploring **site‐specific or disease‐specific damage/injury markers and pathways** with the intent to combine them with functional testing and clinical information. This approach may facilitate an earlier diagnosis in kidney and genitourinary tract diseases and thereby provide a more accurate diagnosis and prognostic assessment, and potentially identify novel routes for intervention. Valuable biomarkers, including uEVs should be linked to mechanistic components of disease processes.

EV‐based biomarkers in urine are currently investigated for an array of malignancies and other diseases such as polycystic kidney disease (Raimondo et al., 2016; Salih et al., 2016), cystinuria (Bourderioux et al., 2015), diabetes (Abe et al., 2018; Lytvyn et al., 2017; Zubiri et al., 2014), renal ischemia‐reperfusion injury (Sonoda et al., 2009), glomerulonephritis (Morikawa et al., 2018), renal interstitial fibrosis (Carreras‐Planella et al., 2020; Chun‐Yan et al., 2018), hypertension or lupus nephritis (Tangtanatakul et al., 2018) and in calcineurin inhibitor‐induced nephrotoxicity (Carreras‐Planella et al., 2020). However, many of the identified candidate biomarkers have not yet been validated in large independent cohorts or tested in more than one laboratory. An exception is the uEV biomarker test for prostate cancer based on PCA3 and ERG that reduces the number of unnecessary prostate biopsies performed (Donovan et al., 2015; McKiernan et al., 2016; McKiernan et al., 2018). Candidate uEV markers require more expansive, multicenter validation, that can provide the large datasets needed to support eventual clinical deployment.

**TEXT BOX 1**

**Characteristics specific to urine and uEVs that influence uEVs analysis**

**Biology**:

- uEVs are (mostly) derived from epithelial cells
- uEVs are (mostly) derived from three major organs: kidney, urothelium, prostate
- Normally, urine does not contain platelets or lipid particles other than EVs
- Urine has variable contamination with microbiota
- Urine composition is highly variable (pH; osmolality, concentration) and influenced by certain medications and diet

**Collection**:

- Urine collection is minimally invasive
- Urine can be collected in large quantities
- Urine collection is sensitive to collection errors by the patients, that is, mid‐stream versus first void; incomplete timed collections, and so forth
- Release of prostate EVs can be stimulated by digital rectal examination (DRE)
- Urine dipstick may be used as an easy quality control of urine
- Urine can contain cells that should (and can easily) be cleared before freezing

**Separation / characterization**:

- Uromodulin lowers yield of uEV separation techniques
- Kidney disease can cause proteinuria/albuminuria and interfere with molecular uEV analysis

**Normalization**:

- An absolute uEV excretion rate can be determined from timed urine collection
